# iMSC-mediated delivery of ACVR2B-Fc fusion protein reduces heterotopic ossification in a mouse model of fibrodysplasia ossificans progressiva

**DOI:** 10.1186/s13287-024-03691-7

**Published:** 2024-03-18

**Authors:** Pan Gao, Yoshiko Inada, Akitsu Hotta, Hidetoshi Sakurai, Makoto Ikeya

**Affiliations:** 1 State Key Laboratory of Oral Diseases and National Center for Stomatology and National Clinical Research Center for Oral Diseases and, Department of General Dentistry, West China Hospital of Stomatology, Sichuan University, Chengdu, 610041 Sichuan China; 2 Department of Clinical Application, Center for iPS Cell Research and Application, Kyoto University, 53, Kawahara-cho, Shogoin, Sakyo-ku, Kyoto, 606-8507 Japan

**Keywords:** Fibrodysplasia ossificans progressiva, Heterotopic ossification, Induced pluripotent stem cells, Mesenchymal stem/stromal cells, ACVR2B-Fc fusion protein

## Abstract

**Background:**

Fibrodysplasia ossificans progressiva (FOP) is a rare genetic disease caused by a gain-of-function mutation in ACVR1, which is a bone morphogenetic protein (BMP) type I receptor. Moreover, it causes progressive heterotopic ossification (HO) in connective tissues. Using FOP patient-derived induced pluripotent stem cells (FOP-iPSCs) and mouse models, we elucidated the underlying mechanisms of FOP pathogenesis and identified a candidate drug for FOP.

**Methods:**

In the current study, healthy mesenchymal stem/stromal cells derived from iPSCs (iMSCs) expressing ACVR2B-Fc (iMSC^ACVR2B-Fc^), which is a neutralizing receptobody, were constructed. Furthermore, patient-derived iMSCs and FOP mouse model (ACVR1^R206H^, female) were used to confirm the inhibitory function of ACVR2B-Fc fusion protein secreted by iMSC^ACVR2B-Fc^ on BMP signaling pathways and HO development, respectively.

**Results:**

We found that secreted ACVR2B-Fc attenuated BMP signaling initiated by Activin-A and BMP-9 in both iMSCs and FOP-iMSCs in vitro. Transplantation of ACVR2B-Fc-expressing iMSCs reduced primary HO in a transgenic mouse model of FOP. Notably, a local injection of ACVR2B-Fc-expressing iMSCs and not an intraperitoneal injection improved the treadmill performance, suggesting compound effects of ACVR2B-Fc and iMSCs.

**Conclusions:**

These results offer a new perspective for treating FOP through stem cell therapy.

**Supplementary Information:**

The online version contains supplementary material available at 10.1186/s13287-024-03691-7.

## Background

Fibrodysplasia ossificans progressiva (FOP) is a rare autosomal-dominant genetic disorder characterized by progressive heterotopic ossification (HO) at extraskeletal sites. A point mutation (c.617G > A; p.R206H) in the *ACVR1* gene (encoding bone morphogenetic protein (BMP) type I receptor, ALK2) was initially identified as the causative origin of classic FOP [1]. The ACVR1^R206H^ mutation accounts for approximately 97% of all the 13 identified variants [2, 3]. ACVR1 is generally known to phosphorylate receptor-specific SMADs (R-SMADs) upon phosphorylation by the BMP type II receptor kinase [4]. Once it forms a heteromeric complex with SMAD4, the complex translocates into the nucleus, which modulates gene transcription by binding the promotor regions of target genes [5]. Gain-of-function mutations in the glycine-and serine-rich (GS) domain of ACVR1^R206H^ impair its autoinhibitory interaction with regulatory protein FKBP12 [6, 7], thus leading to constitutive downstream activation without upstream ligand binding as well as hypersensitivity to native ligands involved in BMP signaling [8]. Ligand-independent BMP signaling still requires type II receptors to provide a nonenzymatic scaffold to form active and functional complexes.

Activins, which are members of the transforming growth factor (TGF)-β family, mediate multiple biological processes by either transducing the TGF-β − SMAD2/3 signaling or regulating other intracellular pathways, including the p38 mitogen-activated protein kinases (p38 MAPK), extracellular signal-regulated kinases 1 and 2 (ERK1/2), and Jun NH_2_-terminal kinase (JNK) pathways [9]. Under normal physiological conditions, Activin-A binds with ACVR1/type II BMP receptor to form a non-signaling complex, which can be transformed into a signaling complex in FOP-mutant ACVR1 [10]. Reportedly, ACVR1^R206H^ aberrantly transduces BMP signaling in response to Activin-A, called neofunction of ACVR1 [11, 12]. Moreover, in our previous work, we found that ACVR2A-Fc and ACVR2B-Fc drastically suppressed Activin-A dependent BMP signaling and enhancement of chondrogenesis in vitro*,* while ACVR1-Fc, BMPR1-Fc, and BMPR2-Fc did not [11]. Hatsell et al. confirmed that recombinant ACVR2A-Fc and ACVR2B-Fc prevented HO formation in an ACVR1^R206H^ mouse model [12]. There have been 13 Fc-fusion drugs approved by the European Union and the United States [13]. However, currently available commercial products utilizing Fc fusion proteins are extremely expensive. Thus, they are not affordable for most patients with FOP, even if they have been approved clinically.

Based on the pluripotency of induced pluripotent stem cells (iPSCs), a broad spectrum of cells that recapitulate hallmarks of intractable diseases can be differentiated from human patient-specific iPSCs [14, 15]. Previously, we generated FOP patient-derived iPSCs (FOP-iPSCs) using FOP dermal fibroblasts via retroviral integration [16]. We found that FOP-iPSCs demonstrated increased chondrogenesis and mineralization capacities in vitro. To obtain genetically matched controls, we established mutation-rescued iPSCs (resFOP-iPSCs) from FOP-iPSCs via bacterial artificial chromosome (BAC)-based homologous recombination technique, in order to correct the *ACVR1*^R206H^ mutation [17]. Mesenchymal stem/stromal cells (MSCs) were established from resFOP-iPSCs (FOP-iMSCs) via a neural crest cell-derived induction method [18], and they demonstrated enhanced chondrogenic capability [17]. Activin-A aberrantly activated ACVR1^R206H^ BMP signaling in vitro and induced endochondral ossification in vivo using FOP-iMSCs [11]*.* Recently, we showed that muscle resident neural crest-derived mesenchymal progenitor cells give rise to HO in mouse models [19]. Consequently, we identified mammalian target of rapamycin (mTOR) signaling as a critical pathway for aberrant FOP-associated chondrogenesis [20]. Notably, a phase 2/3 clinical trial of rapamycin (an mTOR inhibitor) is currently ongoing (UMIN000028429). Collectively, the (res)FOP-iPSCs and (res)FOP-iMSCs allowed for FOP pathogenesis modeling and drug discovery.

MSCs are useful for disease modeling and drug finding and are also considered promising for cell therapy due to their self-renewal, multipotentiality, and functions of immunomodulation and tissue regeneration capacities [21]. Given the disadvantages of donor-associated heterogeneity and finite proliferation of tissue-derived MSCs, induced-MSCs (iMSCs) derived from iPSCs of healthy donors may be a suitable alternative because of their lower heterogeneity [22] and readily scalable capability [23]. We recently developed a thorough xeno-free and highly efficient technique to generate GMP-compatible iMSCs [24]. These iMSCs resemble the tissue-derived MSCs in MSC marker expression, multipotentiality, and global gene expression profiles as well as promote skeletal muscle regeneration of injured muscles. Thus, healthy iMSCs may be useful for cell therapy to treat FOP, wherein soft-tissue injuries always trigger HO.

Although both ACVR2A-Fc and ACVR2B-Fc strongly bind Activin-A to prevent the ligand-receptor binding, ACVR2B-Fc showed higher affinity with BMP-9 [25], which has strong activity in bone formation [26]. Therefore, we focus on ACVR2B-Fc in this study. Herein, we constructed healthy iMSCs generating ACVR2B-Fc fusion protein and elucidated the compound effects of stem cells and therapeutic protein on FOP HO. Our findings indicate that the ACVR2B-Fc fusion protein is a therapeutic drug candidate for the treatment of FOP and that iMSCs can be applied to deliver this therapeutic protein.

## Materials and methods

### Cell culture and reagents

The induction and maintenance of iPSC-derived neural crest cells (iNCCs) and MSCs (iMSCs) were conducted as previously described, with minor modifications [16, 17]. Briefly, iPSCs (1231A3) [27] were maintained in StemFit AK03N (Ajinomoto, Japan) supplemented with 10 µM Y27632 (Wako Pure Chemical, Japan) in dishes pre-coated with iMatrix-511 (nippi, Japan). iNCCs were induced in StemFit Basic03 (equivalent to AK03N without basic fibroblast growth factor (FGF2), Ajinomoto, Tokyo, Japan) with 10 µM SB-431542 (Selleck Chemicals, USA) and 1 µM CHIR99021 (Axon Medchem, Netherlands) for 10 d. iNCCs were maintained in StemFit Basic03 with 10 µM SB-431542 and 20 ng/mL recombinant human FGF2 (WAKO) and recombinant human EGF (R&D Systems, USA) onto plates pre-coated with fibronectin (Merck, USA). iMSCs were induced and maintained in PRIME-XV MSC Expansion XSFM (Fujifilm Irvine Scientific, USA) onto the fibronectin-coated plates. FOP-iMSCs and resFOP-iMSCs were generated as previously described [11, 16] and cultured in αMEM (Invitrogen) supplemented with 5 ng/ml FGF2 (WAKO), 10% fetal bovine serum (FBS; Nichirei, Inc., Japan), and 0.5% penicillin and streptomycin (Invitrogen). The reagents are listed in Additional file 1: Table S1.

### Construction of stably expressing cells

Samples of pPB-CAG-ACVR2B-Fc-His-Puro (5 µg, ACVR2B-Fc) and pPV-EF1a-EGFP-IRES-Puro (5 µg, Control) plasmids were mixed with 1 µg of pHL-EF1a-hcPBase-A plasmid (PBase) in Opti-MEM (Gibco, USA). For IVIS imaging assay, 5 µg of pPV-EF1a-Luciferase-BleoR and 1 µg of PBase plasmids were mixed with 5 µg of pPB-CAG-ACVR2B-Fc-His-Puro and 5 µg of pPV-EF1a-EGFP-IRES-Puro. A total of 100 µl of the DNA and iMSC mixture was obtained. According to the manufacturer's instructions, electroporation was performed using a NEPA21 electroporator (Nepagene, Japan). Stably expressing cells were selected using 1 µg/ml puromycin or 1 µg/ml puromycin (Gibco) with 400 µg/ml zeocin (InvivoGen) for 7 d. FACS analysis for positive MSC markers was performed as previously described [24].

### Quantitative RT-PCR

Total RNA was isolated using an RNeasy Mini Kit (Qiagen, USA) and treated with a DNase-one Kit (Qiagen) to remove any genomic DNA, according to the manufacturer’s instructions. Thereafter, 300 ng RNA was used to perform first-strand cDNA synthesis using SuperScript™ III Reverse Transcriptase (Invitrogen, USA). Quantitative PCR was performed in triplicates using Thunderbird Next SYBR qPCR Mix (TOYOBO, Osaka, Japan) and QuantStudio 3 RealTime PCR System (Applied Biosystems, Forester City, CA, USA). The primer sequences are listed in Additional file 1: Table S2.

### Western blotting analysis

To assess any phosphorylated proteins, iMSC^Control^ and iMSC^ACVR2B-Fc^ cells were starved using 10% XSFM + 90% αMEM with or without 1 µM FK506 for 24 h and treated with the indicated ligands for 1 h. resFOP-iMSC and FOP-iMSC cells were serum-starved using αMEM for 3 h and treated with conditioned medium from iMSC^Control^ or iMSC^ACVR2B-Fc^ (supplemented with the indicated ligands) for 1 h. Protein samples were harvested using RIPA buffer (Wako Fujifilm) supplemented with Halt Protease and Phosphatase Inhibitor Cocktail (Thermo Fisher Scientific). After sonication (Bioruptor), the protein concentrations were detected using Pierce BCA Protein assay reagent (Thermo Fisher Scientific). Next, 10 µg protein was mixed with 6X sample loading buffer (Tokyo Chemical Industry) and incubated at 95 ℃ for 10 min. SDS-PAGE was performed using e-PAGE 10% gel with WSE-1150 Page Run Ace (ATTO, Japan). Blotting was performed using iBlot™ 2 Transfer Stacks (PVDF) with an iBlot™ 2 Gel Transfer Device (Invitrogen). Non-specific antigens were blocked with Blocking One-P (Nacalai Tesque, Japan) for phosphorylated protein analysis and 5% skim milk in TBST for total protein analysis. Phosphorylated antibodies were diluted using Can Get Signal Immunostain Solution B (TOYOBO). All the other antibodies were diluted in 5% skim milk in TBST. Signals were detected with Amersham ECL Prime Western Blotting Detection Reagent (Cytiva) and visualized using an Amersham-ImageQuant-800-biomolecular-imager (Cytiva). Conjugated antibodies on blotting membrane were removed using WB Stripping Solution (Nacalai Tesque). Washed blotting membranes were used for a second antigen–antibody reaction. The antibodies used in western blotting are listed in Additional file 1: Table S3.

### ELISA

Cell culture supernatants were centrifuged at 10,000 × *g* and 4 ℃ for 10 min. His-tagged ACVR2B-Fc proteins were detected using a His-Tag ELISA Detection Kit (L00436, Genscript). Blood samples of mice were left at room temperature (RT, 20–25 ℃) for 30 min until clot formation. The mouse serum was harvested after centrifugation for 15 min at 2,000 × *g*. Activin-A and Fc fragment in serum were detected using Human/Mouse/Rat Activin-A Quantikine ELISA Kit (DAC00B, R&D Systems) and Human Fcγ (Fc Fragment of IgG) ELISA Kit (E-EL-H1745, Elabscience). The absorbances at 450 nM were read using an EnVision Multilabel Reader (PerkinElmer).

### Luciferase assay

Cells were seeded at 1 × 10^5^ per well with 500 μl XSFM medium onto a 24-well plate. The following day, the cells were transiently transfected with plasmids as pGL3-BRE-Luc [28]: pRL-CMV-renilla = 10:1 or pENTR(CAGA)9-Luc2 [11]:pRL-CMV-renilla = 10:1. pRL-CMV-renilla was used as the internal control. FuGene® HD Transfection Reagent (Promega Corporation, USA) was used for transfection for 24 h at a 3:1 ratio to DNA, according to the manufacturer’s instructions. The iMSC^Control^ and iMSC^ACVR2B-Fc^ cells were stimulated with indicated ligands without a medium change. For resFOP-iMSCs and FOP-iMSCs, the medium was replaced with conditioned medium from iMSC^Control^ or iMSC^ACVR2B-Fc^ after 24 h of transfection and supplemented with the indicated ligands. After incubation for 16 h, luciferase activity was measured using a dual luciferase reporter assay system (Promega) on an EnVision® Multilabel Reader (PerkinElmer).

### FOP mouse model

The cardiotoxin-induced ACVR1^R206H^ conditional transgenic mouse model was described previously [20]. Briefly, female mice aged 16 − 21 weeks were administrated with drinking water supplemented with 2 mg/ml doxycycline hyclate (Dox, Sigma Aldrich) and 10 mg/ml sucrose (Nacalai Tesque) to initiate the ACVR1^R206H^ expression. The right gastrocnemius muscle injury was induced via cardiotoxin (CTX, 9.1 μg/mouse; latoxan) injection. Mice were randomly allocated into each group (cage) using a random-number table. For the primary HO model, four injections of 1.5 × 10^6^ cells in 100 µl αMEM were locally or intraperitoneally administered every 5 d using a 27G syringe. For the recurrent HO model, the HO tissues were surgically resected as much as possible under isoflurane anesthesia after 2 weeks of CTX-initiated muscle injury. The wounds were sutured steadily using 5–0 stitches (Bear Medic Corporation, Japan). Three injections of 3 × 10^6^ cells were administered intraperitoneally every 4 d, starting the day after surgery. The mice that died before the endpoint of the experiments were excluded from the analysis. The mouse blood sampling was performed through cardiac puncture under isoflurane anesthesia. Subsequently, specimens were harvested after euthanasia via carbon dioxide inhalation. Our manuscript reporting adheres to the ARRIVE guidelines in accordance with BioMed Central editorial policies.

### In vivo imaging

After injecting 150 mg/kg d-luciferin substrate (Summit Pharmaceutical International Corporation, XLF-1) diluted in PBS for 15 min, mice were anesthetized using isoflurane (Pfizer, USA) and imaged using an IVIS Lumina series III (PerkinElmer). The region of interest was selected to cover all signals, and luminescent intensity was measured using Living Image Software (Caliper Life Sciences, USA).

### Rotarod and treadmill test

After adaptive training, mice were loaded onto a rotarod station (ENV-574 M, Med Associates, USA) and Rodent Treadmill (Ugo Basile SRL, Italy). The rotation speed was set from 2 to 20 rpm and maintained at 20 rpm until the mice fell. The riding times were recorded. The treadmill movement test (+ 5º inclination) started at a 5 m/min velocity, gradually increasing to a maximum of 20 m/min (shock: 1 Hz, shock intensity 0.2 mA). Exhaustion was defined as the mice being shocked 15 times or not returning to the treadmill and staying on the shock grid for 5 s. Running distances were recorded.

### X-ray and micro-computed tomography (μCT) imaging

A mixture of medetomidine, midazolam, and butorphanol was injected intraperitoneally to anesthetize the mice. X-ray images were acquired using an AB-35 system (Acrobio, Japan). The μCT images were scanned using inspeXio SMX100CT (Shimadzu, Kyoto, Japan) and analyzed using FCS-Bon64 (Ratoc System Engineering, Tokyo, Japan) software.

### Histochemical and IHC analyses

Tissue samples from the mice were fixed with 4% paraformaldehyde for 24 h, decalcified in 12% EDTA for 10 d, embedded in paraffin, sectioned, deparaffinized and stained with hematoxylin and eosin (H&E) and Alcian blue. For IHC staining, antigen retrieval was performed via incubation with Liberate Antibody Binding Solution (Polysciences, USA) for 20 min at RT. Non-specific antigens were blocked using Blocking One (Nacalai Tesque) for 1 h at RT. For antibodies generated from the mouse host, tissues were blocked with ReadyProbes™ Mouse on Mouse IgG Blocking Solution (Invitrogen) for 60 min at RT. Antibodies (diluted in Can Get Signal Immunostain Solution B, TOYOBO) were incubated overnight at 4℃. After rinsing in PBST buffer, samples were incubated with Alexa Fluor secondary antibodies diluted in Can Get Signal Immunostain Solution B for 1 h at RT. A mounting medium with DAPI (Vector Laboratories) was used to counterstain the nuclei. Images were captured using a BZ-X810 microscopy (Keyence, Japan). The antibodies for IHC are listed in Additional file 1: Table S3.

### Statistical analysis

The statistical significance of all experiments was calculated using unpaired t-tests, one-way analysis of variance (ANOVA) with Tukey’s multiple comparisons tests, two-way ANOVA with Šídák’s multiple comparisons tests, or two-way ANOVA with Tukey’s multiple comparisons tests. The tests used are indicated in each figure legend. All statistical analyses were performed using GraphPad Prism 9 (GraphPad Software). P values < 0.05 were considered statistically significant. Significance levels are: *P < 0.05; **P < 0.01; ***P < 0.001; ****P < 0.0001.

## Results

### Ligands induced lower BMP and TGF-β signaling in iMSC^ACVR2B-Fc^ than in iMSC^Control^

**Fig. 1:**
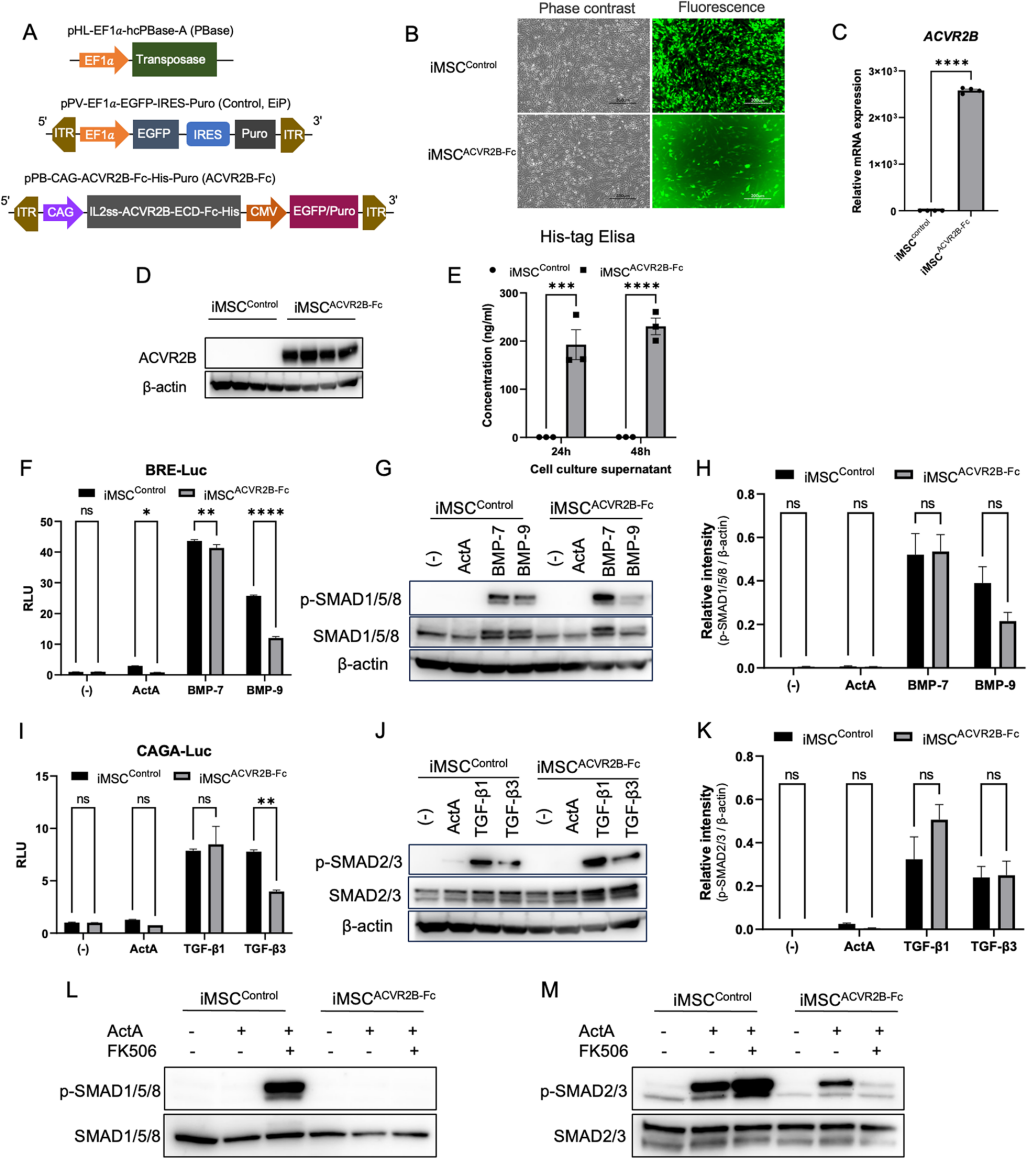
Ligands induced less BMP and TGF-β signaling in iMSC^ACVR2B-Fc^ than in iMSC^Control^. **A** Schematic linear maps of the plasmids used in the study. ITR, inverted terminal repeat; CAG, EF1*a*, CMV: promoter; IRES, internal ribosome entry site. **B** Representative images of stably expressing cells after puromycin selection for 7 d. Scale bar, 300 µm. **C** The relative mRNA levels of *ACVR2B* in iMSC^ACVR2B-Fc^ and control cells based on qPCR analysis. **D** Protein levels of ACVR2B in iMSC^ACVR2B-Fc^ and the control cells based on western blotting. β-actin acts as an internal reference. All full-length blots are presented in Additional file 2: Fig. 1D. **E** The concentration of His-tag proteins in the cell supernatants at two-time points based on ELISA. **F** Activin-A, BMP-7, and BMP-9 induced less BRE-Luc activity in iMSC^ACVR2B-Fc^ than in iMSC^Control^. **G** Representative image of western blotting analysis. BMP-9 induced less phosphorylation of SMAD1/5/8 (p-SMAD1/5/8) in iMSC^ACVR2B-Fc^ than in iMSC^Control^. All full-length blots are presented in Additional file 2: Fig. 1G. **H** Quantification of relative p-SMAD1/5/8 levels normalized to β-actin. **I** TGF-β3 induced less CAGA-Luc activity in iMSC^ACVR2B-Fc^ than in iMSC^Control^. **J** Representative image of western blotting analysis. Activin-A and TGF-β3 induced less phosphorylation of SMAD2/3 (p-SMAD2/3) in iMSC^ACVR2B-Fc^ than in iMSC^Control^. All full-length blots are presented in Additional file 2: Fig. 1 J. **K** Quantification of relative p-SMAD2/3 levels, normalized to β-actin. **L** Representative image of western blotting analysis. FK506 initiated increased Activin-A-dependent activation of BMP signaling in iMSC^Control^, compared with that in iMSC^ACVR2B-Fc^. All full-length blots are presented in Additional file 2: Fig. 1L. **M** ACVR2B-Fc suppressed the Activin-A-dependent activation of TGF-β signaling and inhibited the enhanced activation of TGF-β signaling induced by FK506. All full-length blots are presented in Additional file 2: Fig. 1 M. Activin-A, 100 ng/ml; BMP-7, 100 ng/ml; BMP-9, 10 ng/ml; TGF-β1 & TGF-β3, 10 ng/ml; FK506, 1 µM. Results represent the mean ± SEM. n.s., no significant difference; *, P < 0.05; **, P < 0.01; ***, P < 0.001, ****, P < 0.0001 by unpaired t-test for qPCR and two-way ANOVA with Šídák's multiple comparisons test for others

For stable overexpression of the ACVR2B-Fc fusion protein as a candidate inhibitor of the aberrant signal from the mutated ACVR1^R206H^ receptor, we introduced the ACVR2B-Fc transgene with a His-tag into iMSCs. The plasmids of pPB-CAG-ACVR2B-Fc-His-Puro (ACVR2B-Fc) and PBase or pPV-EF1a-EGFP-IRES-Puro (Control) and PBase (Fig. 1A) were successfully delivered into iMSC by electroporation. Stably expressing cells (iMSC^ACVR2B-Fc^ and iMSC^Control^) were obtained after puromycin (1 µg/ml) selection for 7 d (Fig. 1B). RT-qPCR and Western blotting confirmed the ACVR2B-Fc gene and protein expression (Fig. 1C and D). ELISA for His-tag protein confirmed the secretion of Fc-fusion proteins in the iMSC cell culture supernatant (Fig. 1E). A BMP-specific luciferase reporter construct (BRE-Luc) and TGF-β-responsive luciferase reporter construct (CAGA-Luc) were transfected into iMSC^ACVR2B-Fc^ and iMSC^Control^. Luminescence was detected after ligand stimulation for 16 h. BMP-7, BMP-9, and Activin-A induced lower BRE-Luc activity in iMSC^ACVR2B-Fc^ than in iMSC^Control^ (Fig. 1F). SMAD1/5/8 phosphorylation (stimulated by BMP-9) was lower in iMSC^ACVR2B-Fc^ than in iMSC^Control^ (Fig. 1G). However, the difference was not statistically significant (Fig. 1H). Similarly, Activin-A and TGF-β3 induced lower CAGA-Luc activity in iMSC^ACVR2B-Fc^ than in iMSC^Control^ (Fig. 1I). SMAD2/3 phosphorylation (stimulated by Activin-A) was lower in iMSC^ACVR2B-Fc^ than that in the iMSC^Control^ (Fig. 1J), although this difference was not statistically significant (Fig. 1K). In consistent with our previous report [11], the administration of FK506, an FKBP12 inhibitor, initiated Activin-A-dependent activation of BMP signaling in normal iMSC^Control^ cells, which was also drastically inhibited by ACVR2B-Fc (Fig. 1L). ACVR2B-Fc suppressed the Activin-A-dependent activation of TGF-β signaling and inhibited FK506-mediated activation of TGF-β signaling (Fig. 1M).

### iMSC^ACVR2B-Fc^-conditioned medium inhibited BMP and TGF-β signaling in FOP-iMSCs

**Fig. 2:**
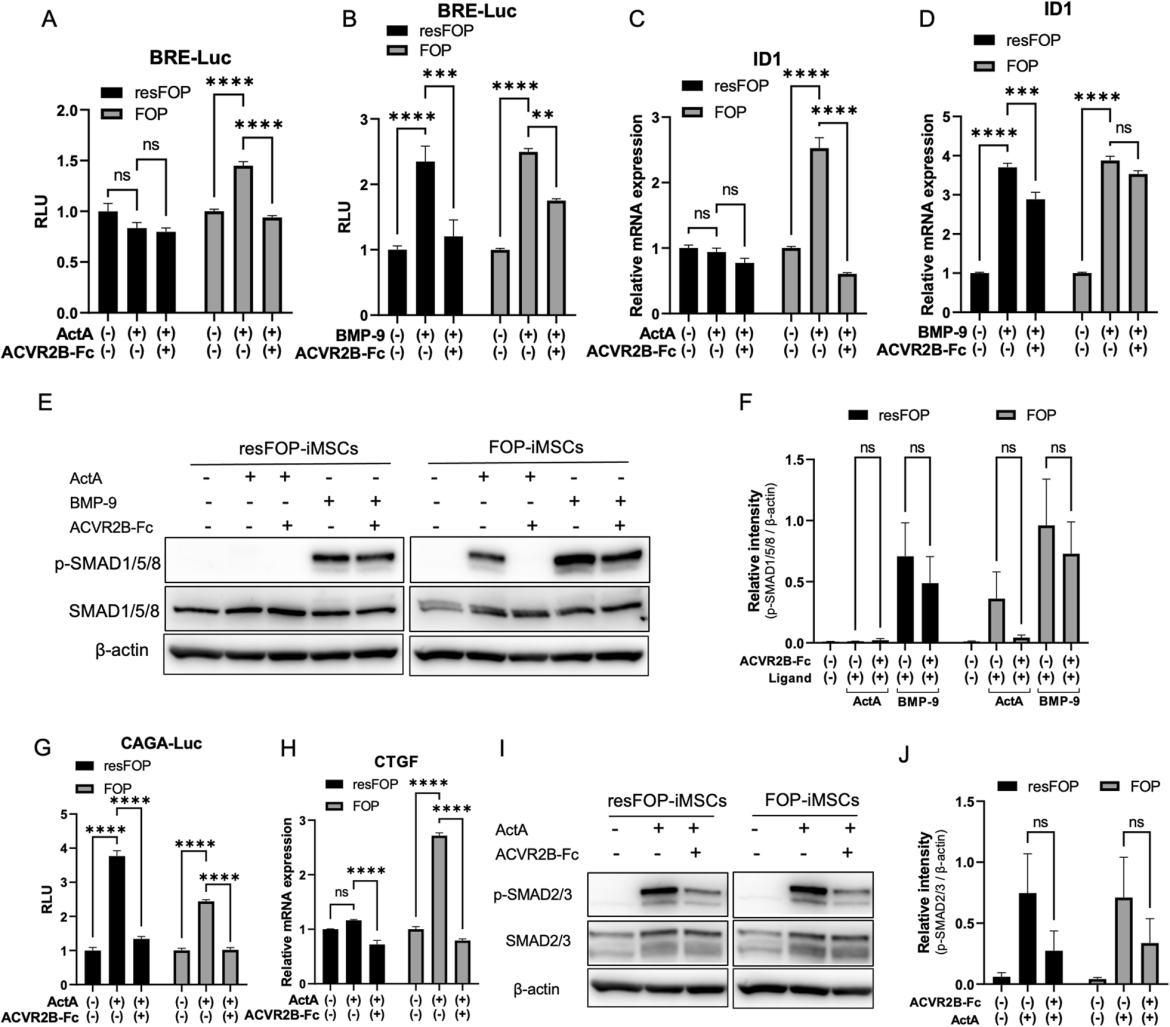
iMSC^ACVR2B-Fc^-conditioned medium inhibited BMP and TGF-β signaling in FOP-iMSCs. **A**, **B** iMSC^ACVR2B-Fc^-conditioned medium inhibited the BRE-Luc activity in FOP-iMSCs (induced by Activin-A and BMP-9) and in resFOP-iMSCs (induced by BMP-9). **C**, **D** iMSC^ACVR2B-Fc^-conditioned medium downregulated the expression of *ID1* (a downstream gene of the BMP pathway) in FOP-iMSCs (induced by Activin-A and BMP-9) and in resFOP-iMSCs (induced by BMP-9). **E** Representative image of western blotting analysis. iMSC^ACVR2B-Fc^-conditioned medium inhibited SMAD1/5/8 phosphorylation (induced by Activin-A and BMP-9) in FOP-iMSCs. All full-length blots are presented in Additional file 2: Fig. 2E. **F** Quantification of relative p-SMAD1/5/8 levels, normalized to β-actin. **G** iMSC^ACVR2B-Fc^-conditioned medium inhibited the CAGA-Luc activity (induced by Activin-A) in resFOP-iMSCs and FOP-iMSCs. **H** iMSC^ACVR2B-Fc^-conditioned medium downregulated the expression of *CTGF* (a downstream gene of the TGF-β pathway; induced by Activin-A) in FOP-iMSCs. **I** Representative image of western blotting analysis. iMSC^ACVR2B-Fc^-conditioned medium inhibited SMAD2/3 phosphorylation (induced by Activin-A) in FOP-iMSCs. All full-length blots are presented in Additional file 2: Fig. 2I. **F** Quantification of relative p-SMAD2/3 levels, normalized to β-actin. Results represent the mean ± SEM. **, P < 0.01; ***, P < 0.001; ****, P < 0.0001 by two-way ANOVA with Šídák's multiple comparisons test. Activin-A, 100 ng/ml; BMP-9, 10 ng/ml

As previously described [16, 17], FOP-iMSCs and resFOP-iMSCs were generated from FOP patient-derived iPSCs (FOP-iPSCs) and gene-corrected iPSC clones (rescued via BAC-based homologous recombination) through a neural crest cell lineage. After transfection of BRE-Luc or CAGA-Luc reporters, cells were treated with the conditioned medium from iMSC^ACVR2B-Fc^ or iMSC^Control^ supplemented with Activin-A or BMP-9. In accordance with previous reports [11], Activin-A aberrantly transduced BMP signaling in FOP-iMSCs other than resFOP-iMSCs (Fig. 2A). Conditioned medium from iMSC^ACVR2B-Fc^ reduced the activation of BMP signaling stimulated by Activin-A in FOP-iMSCs (Fig. 2A). BMP-9 activated the BMP signaling in both resFOP-iMSCs and FOP-iMSCs, and this activation was inhibited by conditioned medium from iMSC^ACVR2B-Fc^ (Fig. 2B). The expression of *ID1*, a downstream gene of the BMP pathway, was activated by Activin-A and BMP-9 in FOP-iMSCs and downregulated by iMSC^ACVR2B-Fc^ conditioned medium (Fig. 2C and D). The phosphorylation of SMAD1/5/8 (stimulated by Activin-A and BMP-9 in FOP-iMSCs) was inhibited by the conditioned medium from iMSC^ACVR2B-Fc^ (Fig. 2E), although this difference was not statistically significant (Fig. 2F). Conditioned medium from iMSC^ACVR2B-Fc^ also reduced the activation of TGF-β signaling, which was stimulated by Activin-A in FOP-iMSCs and resFOP-iMSCs (Fig. 2G). The expression of *CTGF*, a downstream gene of the TGF-β pathway, was downregulated by iMSC^ACVR2B-Fc^ conditioned medium in FOP-iMSCs (Fig. 2H). The phosphorylation of SMAD2/3 (stimulated by Activin-A in both resFOP-iMSCs and FOP-iMSCs) was inhibited by the conditioned medium from iMSC^ACVR2B-Fc^ (Fig. 2I), although this difference was not statistically significant (Fig. 2J).

### Administration of iMSC^ACVR2B-Fc^ reduced primary HO in a mouse model of FOP

**Fig. 3:**
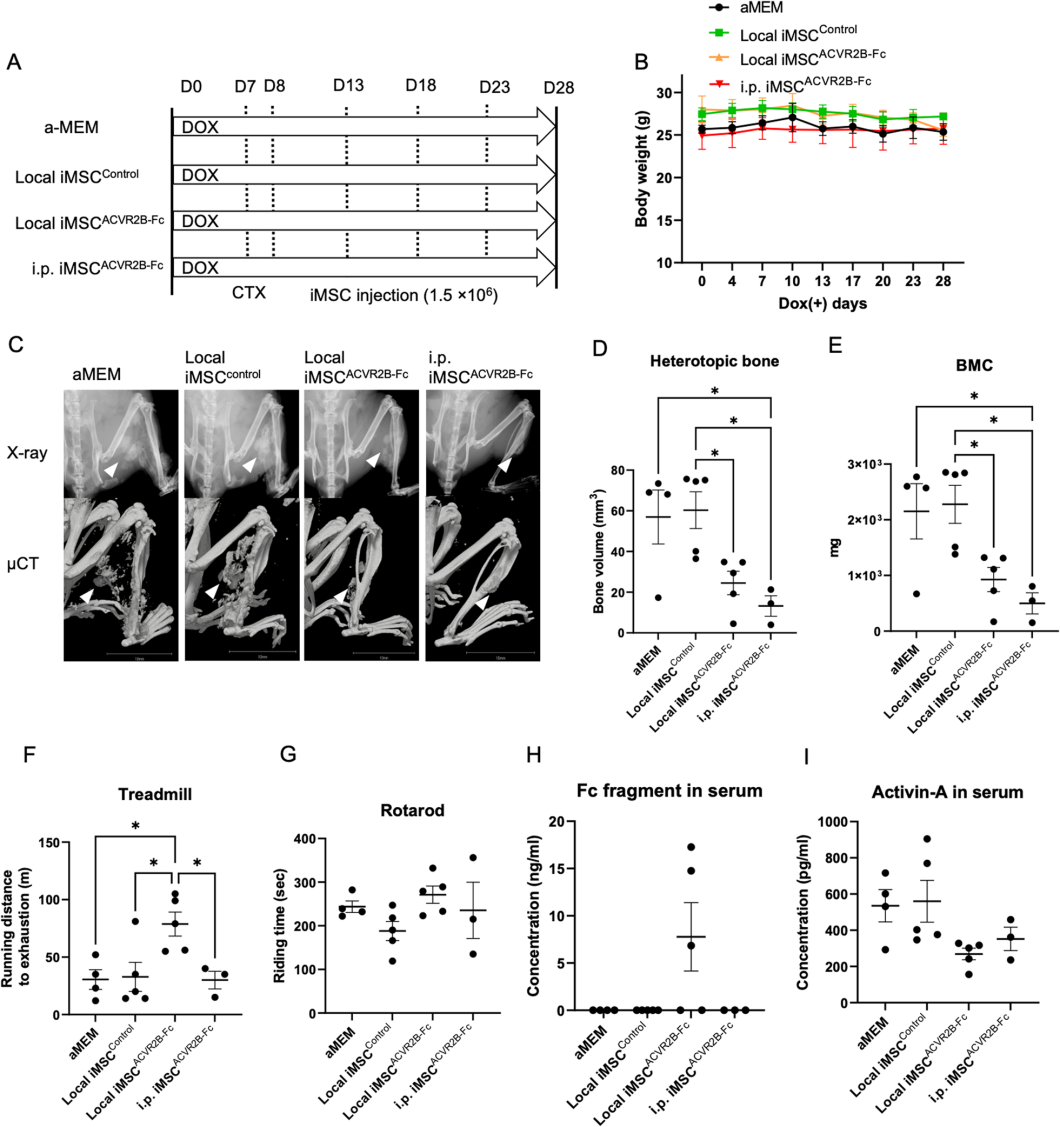
Administration of iMSC^ACVR2B-Fc^ reduced primary HO in a mouse model of FOP. **A** Schematic view of the experiments. CTX was injected on day 7. Cells (1.5 × 10^6^) were administrated on days 8, 13, 18, and 23. **B** Body weight monitoring. **C** Representative X-ray and μCT images of each group. Arrowheads indicate HO. Scale bar, 10 mm. **D** Average of HO volume on day 28. **E** Bone mineral content (BMC) of HO sites. **F** Endurance tests were conducted using a rodent treadmill. **G** The exercise capacities were analyzed using the rotarod test. **H**, **I** Serum Fc fragment and Activin-A levels were detected by ELISA. Results represent the mean ± SEM. *, P < 0.05 by one-way ANOVA with Tukey’s multiple comparison test

**Fig. 4:**
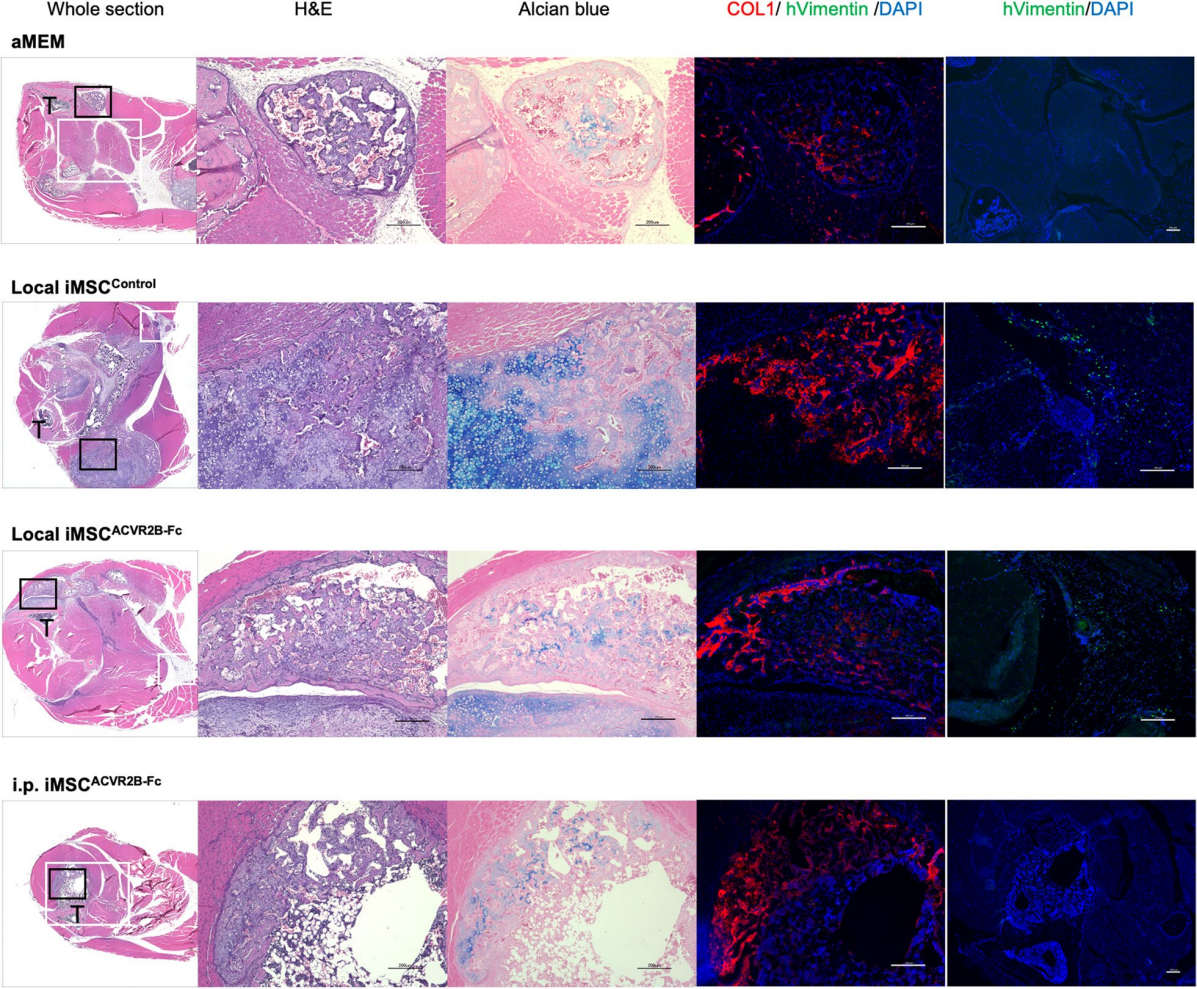
Histological analysis of HO sites. H&E staining, Alcian blue (cartilage), anti-COL1 (bone marker), and anti-hVimentin (transplanted iMSCs) staining are shown. T, Tibia. Black frame, the location of H&E, Alcian blue, and COL1/hVimentin/DAPI staining; white frame, the location of hVimentin/DAPI staining. Scale bar, 200 µm

Luciferase-expressing iMSC^ACVR2B-Fc^ (iMSC^2B−Fc/Luci^) and iMSC^Control^ (iMSC^EiP/Luci^) were obtained after selection via puromycin (1 µg/ml) and zeocin (400 µg/ml) for 7 d (Additional file 3: Fig. S1 A and B). The levels of the *ACVR2B* and *Luciferase* genes, ACVR2B protein, and His-tag protein were confirmed by RT-qPCR, western blotting, and ELISA (Additional file 3: Fig. S1C–F). Stably expressing cells were still positive for MSC makers, including CD44, CD73, CD90, and CD105 (Additional file 3: Fig. S1G and H). To initiate the in vivo experiments, Dox was administered to FOP mice from day 0 to activate ACVR1^R206H^ expression. One week later, CTX was injected into the right gastrocnemius muscle to initiate the muscle injury (Fig. 3A). The body weights of the mice remained relatively stable (Fig. 3B). From day 8, 1.5 × 10^6^ of the indicated iMSCs were transplanted into the CTX-injected area or intraperitoneally (i.p.). IVIS imaging was performed (Fig. S2A); the total intensity of the luciferase signal decreased with time, becoming undetectable by day 5 (Additional file 4: Fig. S2B and C). Thus, four injections of iMSCs were administered every 5 d. On day 28, the X-ray and μCT scans were obtained under general anesthesia (Fig. 3C). The bone volume quantification results showed that local (n = 5) or i.p. (n = 3) administration of iMSC^ACVR2B-Fc^ inhibited HO, compared with local injection of αMEM (n = 4) or iMSC^Control^ (n = 5) (Fig. 3D). The bone mineral content (BMC) of HO was consistent with the HO volume (Fig. 3E). The mice administered with local iMSC^ACVR2B-Fc^ ran the longest distance on the treadmill (Fig. 3F). The rotarod riding time results indicated no differences between the groups (Fig. 3G). ELISA confirmed the existence of ACVR2B-Fc protein in the serum of partial mice administered with iMSC^ACVR2B-Fc^ (Fig. 3H). ELISA results also showed that local or i.p. administration of iMSC^ACVR2B-Fc^ decreased the serum Activin-A levels in mice, compared with the controls, although this difference was not statistically significant (Fig. 3I). In the HO site, positive staining for Alcian blue (pH 1.0, sulfated polysaccharides in cartilage tissues, and COL1 (bone marker) was observed, indicating the occurrence of endochondral ossification (Fig. 4). Anti-human-specific vimentin antibody (hVimentin, cytoskeleton-staining) was used to track the injected iMSCs. The COL1-positive area did not co-stain with hVimentin, implying that HO derived from ACVR1^R206H^ mice rather than differentiated from injected iMSCs (Fig. 4 and Additional file 5: Fig. S3A).

### Administration of iMSC^ACVR2B-Fc^ slightly inhibited recurrent HO in the surgical mouse model of FOP

**Fig. 5:**
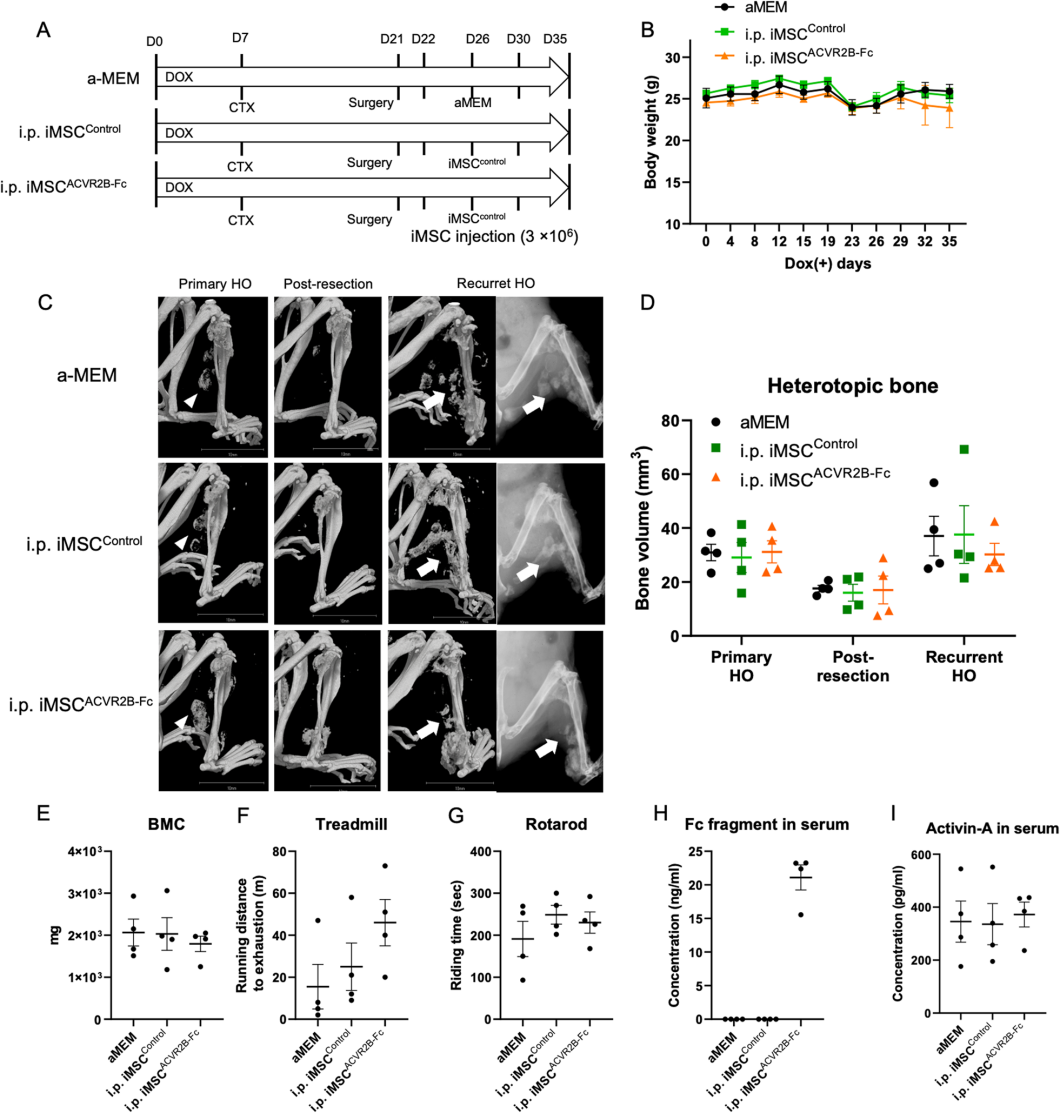
Administration of iMSC^ACVR2B-Fc^ slightly inhibited recurrent HO in the surgical mouse model of FOP. **A** Schematic view of the experiments. CTX was injected on day 7. Primary HO was surgically removed on day 21. Cells (3 × 10^6^) were administrated on days 22, 26, and 30. **B** Body weight monitoring. **C** Representative X-ray and μCT images of each group. μCT images were from pre- and post-surgery and on day 35. Arrowheads indicate primary HO, and arrows indicate recurrent HO. Scale bar, 10 mm. **D** Quantification of primary, post-resected, and recurrent HO. **E** BMC of HO sites. **F** Endurance tests were conducted using a rodent treadmill. **G** The mice’s exercise capacities were analyzed using the rotarod test. **H**, **I** Serum Fc fragment and Activin-A levels were detected by ELISA. Results represent the mean ± SEM. P > 0.05 by one-way ANOVA with Tukey’s multiple comparison test

**Fig. 6:**
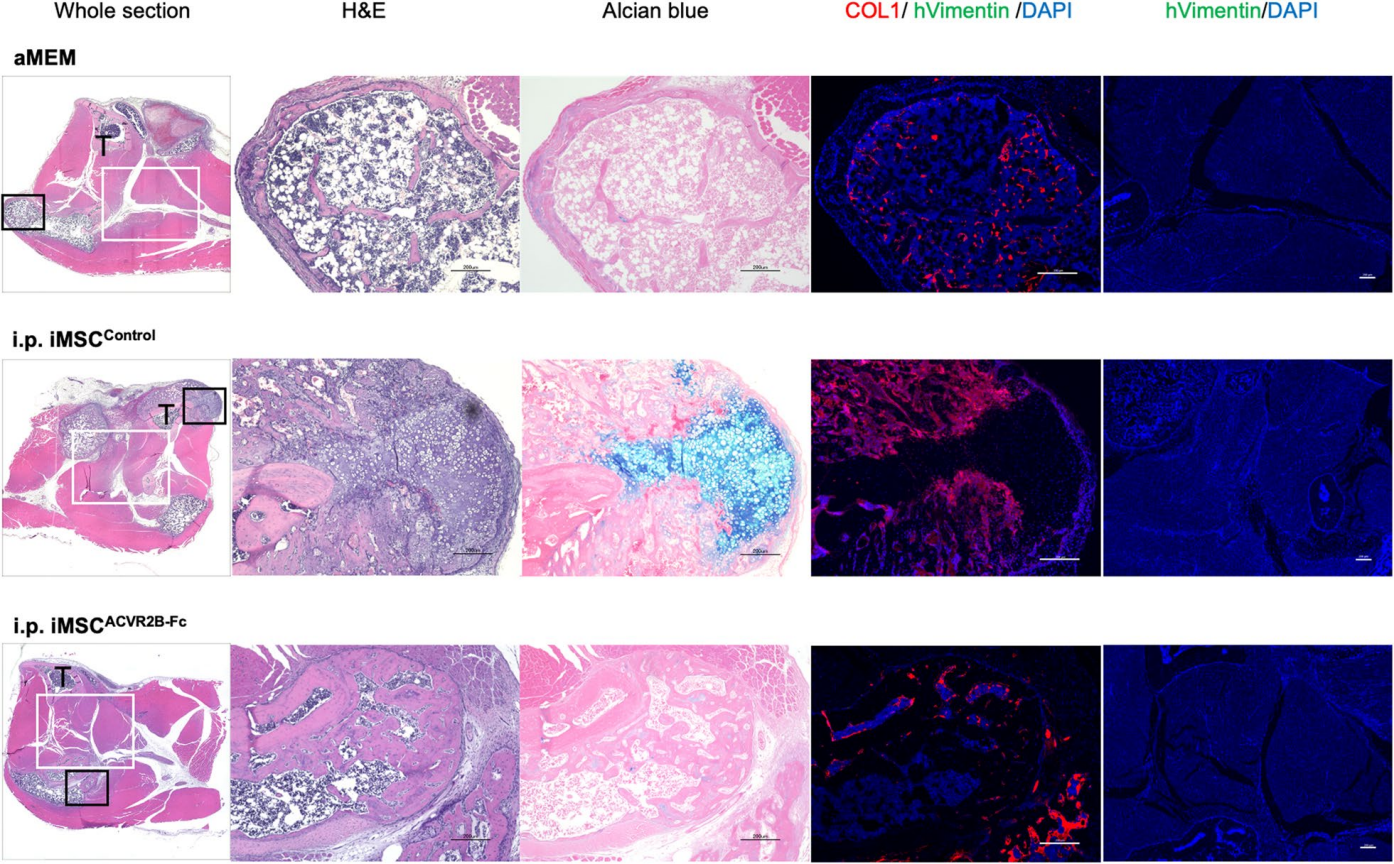
Histological analysis of HO sites. H&E staining, Alcian blue (cartilage), anti-COL1 (bone marker), and anti-hVimentin (transplanted iMSCs) staining are shown. T, Tibia. Black frame, the location of H&E, Alcian blue, and COL1/hVimentin/DAPI staining; white frame, the location of hVimentin/DAPI staining. Scale bar, 200 µm

Aggressive surgical procedures, including HO resection, have been thought to be contraindicated for patients with FOP due to absolute recurrence at the surgical site and systematic disease exacerbation [29]. We established a surgical FOP mouse model to investigate the effects of iMSC^ACVR2B-Fc^ on inhibiting recurrent HO. On day 21, CTX-induced HO tissues were recorded by μCT and were resected as much as possible. The volume of residual HO just after the surgery was also evaluated with μCT. The mice were then divided into three groups (n = 4) of those injected with the following: (1) αMEM (blank control), (2) iMSC^Control^, and (3) iMSC^ACVR2B-Fc^. Considering leakage from the sutured wounds, three injections of the respective vehicle or cells (3 × 10^6^) were administered every 4 d by intraperitoneal injection (i.p.) from day 22 (Fig. 5A). The body weights of the mice remained relatively stable; however, they did decrease, likely due to stress from the surgery (Fig. 5B). On day 35, all the mice were sacrificed. Recurrent HO was evaluated by μCT and X-ray (Fig. 5C). The volume of HO before resection and residual HO was approximately the same among the three groups, with no significant differences. On day 35, mice that received iMSC^Control^ showed a similar volume of recurrent HO as that of the blank control group. Mice that received iMSC^ACVR2B-Fc^ showed a slightly decreased volume of recurrent HO as compared to the mice in the other two groups, although this difference was not statistically significant (Fig. 5D). The BMC of the final HO was similar to the recurrent HO volume (Fig. 5E). Mice that received iMSC^ACVR2B-Fc^ ran the longest distance on the treadmill and demonstrated moderate persistence on the rotarod, although these differences were not statistically significant (Fig. 5F and G). ELISA confirmed the existence of ACVR2B-Fc protein in the serum of mice administered with iMSC^ACVR2B-Fc^ (Fig. 3H). There were no significant differences in the serum Activin-A levels between the three groups (Fig. 5I). At the HO sites, positive staining for Alcian blue and COL1 was observed, indicating the occurrence of endochondral ossification (Fig. 6). At the surgery site, negative staining for hVimentin indicated that none of the injected iMSCs homed to the injured site. The COL1-positive area did not co-stain with hVimentin, implying that HO was also derived from ACVR1^R206H^ mice rather than differentiating from the injected iMSCs (Fig. 6 and Additional file 5: Fig. S3B).

## Discussion

At least two options exist for the prevention of the binding of ligands to their receptors i.e., soluble decoy receptors and neutralizing antibodies. Fc-fusion proteins genetically fuse the fragment crystallizable (Fc) domain of an immunoglobulin G (IgG) to a ligand, active peptide, or extracellular domain (ECD) of a receptor to perform antibody-like functions. It improves the properties of proteins, including their serum half-life, stability, and solubility, and facilitates the use of simplified downstream purification procedures [30]. In the current study, we constructed IL2ss-ACVR2B-ECD-Fc-His (ACVR2B-Fc)-expressing cells using the *piggyBac* transposon system through electroporation (Fig. 1A–E). Expectedly, the secreted ACVR2B-Fc reduced BMP and TGF-β signaling (stimulated by Activin-A) in iMSCs (Fig. 1F–M). Activin-A aberrantly transduced BMP signaling in FOP-iMSCs, but not in resFOP-iMSCs (Fig. 2A, C, E, F). BMP-9 has been shown to induce MSCs to differentiate into adipose, cartilage, and bone tissues [28, 29] by specifically activating BMP signaling [33] with the strongest osteogenic potential of all known BMPs [31, 32]. Similarly, BMP-9 activated BMP signaling in both resFOP-iMSCs and FOP-iMSCs (Fig. 2B and D). iMSC^ACVR2B-Fc^-conditioned medium inhibited the activation of BMP and TGF-β signaling (stimulated by Activin-A and BMP-9) in FOP-iMSCs (Fig. 2). These results suggest that ACVR2B-Fc (produced by healthy iMSCs) can competitively combine Activin-A and BMP-9 and suppress FOP-iMSCs-induced ligand-dependent activation of BMP and TGF-β signaling pathways.

Garetosmab is a monoclonal antibody that selectively binds and blocks signaling transduced by Activins A, AB; further, AC and is currently being evaluated a phase 2 clinical study (NCT04577820) [36]. However, antibodies against ACVR1 enhanced HO in FOP mice by the formation of antibody-mediated dimerization of ACVR1 [37] and dysregulation of the growth of fibro–adipogenic progenitors (FAPs) caused by ACVR1 antibody-induced receptor agonist activity [38]. Compared with monoclonal antibodies, ACVR2B-Fc fusion protein does not selectively combine with ligands that are only involved in the FOP-associated BMP signaling pathway. This non-specific trapping effect may lead to dysfunction of normal physiological processes related to BMP or TGF-β signaling pathways.

FOP flare-ups are known to be triggered by soft-tissue injuries, including accidental trauma and surgical interventions [3]. Surgical procedure is not feasible to remove FOP HO since the resulting injury causes a far more serious HO locally and systematically. Thus, strategies that inhibit inflammation or promote tissue regeneration may alleviate HO development. Although we found that iMSC transplantation can promote the regeneration of skeletal muscles via paracrine factors in NSG mice [24], locally or intraperitoneally transplanted iMSC^Control^ did not prevent primary and recurrent HO in FOP mice (Fig. 3C and D; 5C and D). Nevertheless, local and i.p. transplantation of ACVR2B-Fc-expressing iMSCs reduced the occurrence of primary HO (Fig. 3C and D). The inhibitory effect of i.p. injection seems stronger than local injection, suggesting that the latter induced additional injury in the muscle. Of note, the local injection of iMSC^ACVR2B-Fc^ cells, not iMSC^Control^ or i.p. iMSC^ACVR2B-Fc^, improved the treadmill performance of FOP mice (Fig. 3F), which indicated that local inhibition of HO inhibited by ACVR2B-Fc combined with the muscle fiber regeneration induced by iMSCs can benefit the treadmill performance of mice, suggesting a combined effect of ACVR2B-Fc and iMSCs. However, it is difficult to evaluate muscle fiber regeneration via histological analysis in the HO sites since most of the muscles were replaced by heterotopic bones (Fig. 4). We observed that hVimentin^+^ iMSCs were not present at the COL1^+^ HO sites and vice versa (Fig. 4), indicating that improvement of the survival rate of transplanted iMSCs may prevent more HO formation.

Intraperitoneal transplantation of iMSC^ACVR2B-Fc^ also prevented recurrent HO, although this finding was not statistically significant (Fig. 5C and D). In the primary HO model, locally and intraperitoneally administered iMSC^ACVR2B-Fc^ cells decreased serum Activin-A levels (Fig. 3H), but no for the surgically recurrent situation (Fig. 5H). This may be due to the abundant ligands (of the TGF-β family) generated by inflammatory cells after the surgical procedure. In addition, the transplanted cells may not have been able to maintain effective homeostasis and metabolism to produce sufficient ACVR2B-Fc fusion protein, due to both immune rejection from the host and the surgery-induced cytokine storm. In the future, HLA-genome-edited iPSCs may be a promising cell resource to improve the immune compatibility of iMSCs in human applications [39].

## Conclusions

Although it is difficult to evaluate the merit of the cost burden between purified Fc fusion protein or generated one by iMSCs at the present stage, this study offers a novel viewpoint for treating FOP. Off-the-shelf iMSCs that generate Fc fusion proteins provide a new method to treat FOP through stem cell therapy.

### Supplementary Information

**Additional file 1:** **Supplementary table 1.** Reagents used in cell culture. **Supplementary table 2.** Primer sequences of genes in RT-qPCR. **Supplementary table 3.** Antibodies used in western blotting, IHC, and FACS analyses.

**Additional file 2:** Raw data of Western blotting.

**Additional file 3:** **Fig. S1. **Luciferase-expressing iMSCs. **A** Schematic linear maps of the luciferase plasmid used in the study. **B** Representative images of stably expressing cells after puromycin and zeocin selection for 7 d. iMSC^EiP/Luci^ was used as a control. Scale bar, 200 µm. **C**, **D** The relative mRNA levels of *ACVR2B* and *Luciferase* in the iMSC^2B-Fc/Luci^ and iMSC^EiP/Luci^ cells based on qPCR analysis. **E** Protein levels of ACVR2B in the iMSC^2B-Fc/Luci^ and control cells based on western blotting analysis. β- was used as an internal reference. All full-length blots are presented in Additional file 1: Fig. S1E. **F** The concentration of His-tag proteins in the cell supernatants at two-time points based on ELISA. **G**, **H** The expression of MSC-related markers in iMSC^EiP/Luci^ and iMSC^2B-Fc/Luci^ (dark gray) and isotype control (gray) cells. Results represent the mean ± SEM. **, P < 0.01; ****, P < 0.0001 by one-way ANOVA with Turkey’s multiple comparison test for qPCR and two-way ANOVA with Šídák's multiple comparisons test for ELISA.

**Additional file 4:** **Fig. S2. **Tracking of donor cells after intraperitoneal and local transplantation. **A** In vivo imaging of donor cells within 5 d. CTX was injected into the right gastrocnemius muscle to initiate muscle injury. For the left mouse, cells were transplanted intraperitoneally. For the right mouse, cells were transplanted locally on the CTX-injected site. **B**, **C** The time course of the luciferase signal intensity of two administrations. The signal disappeared on day 5.

**Additional file 5:**  **Fig. S3.** Overlay of the immunofluorescence staining. **A** Co-staining of COL1, hVimentin, and DAPI in Figure 4, lane 4. **B** Co-staining of COL1, hVimentin, and DAPI in Figure 6, lane 4. Scale bar, 200 µm.

## Data Availability

Data available on request.

## Abbreviations

FOP: Fibrodysplasia ossificans progressiva
HO: Heterotopic ossification
iPSCs: Induced pluripotent stem cells
resFOP-iPSCs: Mutation-rescued iPSCs
BAC: Bacterial artificial chromosome
MSCs: Mesenchymal stem/stromal cells
mTOR: Mammalian target of rapamycin
iNCCs: IPSC-derived neural crest cells
FGF2: Basic fibroblast growth factor/fibroblast growth factor 2
FBS: Fetal bovine serum
RT: Room temperature
CTX: Cardiotoxin
μCT: Micro-computed tomography
H&E: Hematoxylin and eosin
ANOVA: Analysis of variance
BMC: Bone mineral content
R-SMADs: Receptor-specific SMADs
GS: Glycine-and serine-rich
TGF: Transforming growth factor
MAPK: Mitogen-activated protein kinase
JNK: Jun NH_2_-terminal kinase
ERK1/2: Extracellular signal-regulated kinase 1 and 2
FAP: Fibro–adipogenic progenitor
ECD: Extracellular domain
Fc: Fragment crystallizable

## References

[1] Shore EM, Xu M, Feldman GJ, Fenstermacher DA, Cho T-J, Choi IH (2006). A recurrent mutation in the BMP type I receptor ACVR1 causes inherited and sporadic fibrodysplasia ossificans progressiva. Nat Genet.

[2] Katagiri T, Tsukamoto S, Kuratani M (2018). Heterotopic bone induction via BMP signaling: potential therapeutic targets for fibrodysplasia ossificans progressiva. Bone.

[3] Ventura F, Williams E, Ikeya M, Bullock AN, Ten Dijke P, Goumans M-J (2021). Challenges and opportunities for drug repositioning in fibrodysplasia ossificans progressiva. Biomedicines.

[4] Agnew C, Ayaz P, Kashima R, Loving HS, Ghatpande P, Kung JE (2021). Structural basis for ALK2/BMPR2 receptor complex signaling through kinase domain oligomerization. Nat Commun.

[5] Massagué J (2012). TGFβ signalling in context. Nat Rev Mol Cell Biol.

[6] Taylor KR, Vinci M, Bullock AN, Jones C (2014). ACVR1 mutations in DIPG: lessons learned from FOP. Cancer Res.

[7] Pacifici M, Shore EM (2016). Common mutations in ALK2/ACVR1, a multi-faceted receptor, have roles in distinct pediatric musculoskeletal and neural orphan disorders. Cytokine Growth Factor Rev.

[8] Bagarova J, Vonner AJ, Armstrong KA, Börgermann J, Lai CSC, Deng DY (2013). Constitutively active ALK2 receptor mutants require type II receptor cooperation. Mol Cell Biol.

[9] Bloise E, Ciarmela P, Dela Cruz C, Luisi S, Petraglia F, Reis FM (2019). Activin A in Mammalian Physiology. Physiol Rev.

[10] Aykul S, Corpina RA, Goebel EJ, Cunanan CJ, Dimitriou A, Kim HJ (2020). Activin A forms a non-signaling complex with ACVR1 and type II Activin/BMP receptors via its finger 2 tip loop. Elife.

[11] Hino K, Ikeya M, Horigome K, Matsumoto Y, Ebise H, Nishio M (2015). Neofunction of ACVR1 in fibrodysplasia ossificans progressiva. Proc Natl Acad Sci USA.

[12] Hatsell SJ, Idone V, Wolken DMA, Huang L, Kim HJ, Wang L (2015). ACVR1R206H receptor mutation causes fibrodysplasia ossificans progressiva by imparting responsiveness to activin A. Sci Transl Med.

[13] Duivelshof BL, Murisier A, Camperi J, Fekete S, Beck A, Guillarme D (2021). Therapeutic Fc-fusion proteins: current analytical strategies. J Sep Sci.

[14] Shi Y, Inoue H, Wu JC, Yamanaka S (2017). Induced pluripotent stem cell technology: a decade of progress. Nat Rev Drug Discov.

[15] Rowe RG, Daley GQ (2019). Induced pluripotent stem cells in disease modelling and drug discovery. Nat Rev Genet.

[16] Matsumoto Y, Hayashi Y, Schlieve CR, Ikeya M, Kim H, Nguyen TD (2013). Induced pluripotent stem cells from patients with human fibrodysplasia ossificans progressiva show increased mineralization and cartilage formation. Orphanet J Rare Dis.

[17] Matsumoto Y, Ikeya M, Hino K, Horigome K, Fukuta M, Watanabe M (2015). New protocol to optimize iPS cells for genome analysis of fibrodysplasia ossificans progressiva. Stem Cells.

[18] Fukuta M, Nakai Y, Kirino K, Nakagawa M, Sekiguchi K, Nagata S (2014). Derivation of mesenchymal stromal cells from pluripotent stem cells through a neural crest lineage using small molecule compounds with defined media. PLoS ONE.

[19] Zhao C, Inada Y, Sekiguchi K, Hino K, Nishio M, Yamada Y (2023). Myelin protein zero (P0)- and Wnt1-Cre marked muscle resident neural crest-derived mesenchymal progenitor cells give rise to heterotopic ossification in mouse models. Genes Dis.

[20] Hino K, Horigome K, Nishio M, Komura S, Nagata S, Zhao C (2017). Activin-A enhances mTOR signaling to promote aberrant chondrogenesis in fibrodysplasia ossificans progressiva. J Clin Invest.

[21] Galipeau J, Sensébé L (2018). Mesenchymal stromal cells: clinical challenges and therapeutic opportunities. Cell Stem Cell.

[22] Zhang J, Chen M, Liao J, Chang C, Liu Y, Padhiar AA (2021). Induced pluripotent stem cell-derived mesenchymal stem cells hold lower heterogeneity and great promise in biological research and clinical applications. Front Cell Dev Biol.

[23] Zhao Q, Gregory CA, Lee RH, Reger RL, Qin L, Hai B (2015). MSCs derived from iPSCs with a modified protocol are tumor-tropic but have much less potential to promote tumors than bone marrow MSCs. Proc Natl Acad Sci USA.

[24] Kamiya D, Takenaka-Ninagawa N, Motoike S, Kajiya M, Akaboshi T, Zhao C (2022). Induction of functional xeno-free MSCs from human iPSCs via a neural crest cell lineage. Npj Regen Med.

[25] Aykul S, Martinez-Hackert E (2016). Transforming growth factor-β family ligands can function as antagonists by competing for type II receptor binding*. J Biol Chem.

[26] Zhou Y-M, Yang Y-Y, Jing Y-X, Yuan T-J, Sun L-H, Tao B (2020). BMP9 reduces bone loss in ovariectomized mice by dual regulation of bone remodeling. J Bone Miner Res.

[27] Nakagawa M, Taniguchi Y, Senda S, Takizawa N, Ichisaka T, Asano K (2015). A novel efficient feeder-free culture system for the derivation of human induced pluripotent stem cells. Sci Rep.

[28] Korchynskyi O, ten Dijke P (2002). Identification and functional characterization of distinct critically important bone morphogenetic protein-specific response elements in the Id1 promoter. J Biol Chem.

[29] Hwang CD, Pagani CA, Nunez JH, Cherief M, Qin Q, Gomez-Salazar M (2022). Contemporary perspectives on heterotopic ossification. JCI Insight.

[30] Levin D, Golding B, Strome SE, Sauna ZE (2015). Fc fusion as a platform technology: potential for modulating immunogenicity. Trends Biotechnol.

[31] Kang Q, Song W-X, Luo Q, Tang N, Luo J, Luo X (2009). A comprehensive analysis of the dual roles of BMPs in regulating adipogenic and osteogenic differentiation of mesenchymal progenitor cells. Stem Cells Dev.

[32] Lamplot JD, Qin J, Nan G, Wang J, Liu X, Yin L (2013). BMP9 signaling in stem cell differentiation and osteogenesis. Am J Stem Cells.

[33] Mostafa S, Pakvasa M, Coalson E, Zhu A, Alverdy A, Castillo H (2019). The wonders of BMP9: From mesenchymal stem cell differentiation, angiogenesis, neurogenesis, tumorigenesis, and metabolism to regenerative medicine. Genes Dis.

[34] Luo Q, Kang Q, Si W, Jiang W, Park JK, Peng Y (2004). Connective tissue growth factor (CTGF) is regulated by Wnt and bone morphogenetic proteins signaling in osteoblast differentiation of mesenchymal stem cells. J Biol Chem.

[35] Sharff KA, Song W-X, Luo X, Tang N, Luo J, Chen J (2009). Hey1 basic helix-loop-helix protein plays an important role in mediating BMP9-induced osteogenic differentiation of mesenchymal progenitor cells. J Biol Chem.

[36] Vanhoutte F, Liang S, Ruddy M, Zhao A, Drewery T, Wang Y (2020). Pharmacokinetics and pharmacodynamics of garetosmab (Anti-Activin A): results from a first-in-human phase 1 study. J Clin Pharmacol.

[37] Aykul S, Huang L, Wang L, Das NM, Reisman S, Ray Y (2022). Anti-ACVR1 antibodies exacerbate heterotopic ossification in fibrodysplasia ossificans progressiva (FOP) by activating FOP-mutant ACVR1. J Clin Invest.

[38] Lees-Shepard JB, Stoessel SJ, Chandler JT, Bouchard K, Bento P, Apuzzo LN (2022). An anti-ACVR1 antibody exacerbates heterotopic ossification by fibro–adipogenic progenitors in fibrodysplasia ossificans progressiva mice. J Clin Invest.

[39] Xu H, Wang B, Ono M, Kagita A, Fujii K, Sasakawa N (2019). Targeted disruption of HLA genes via CRISPR-Cas9 generates iPSCs with enhanced immune compatibility. Cell Stem Cell.

