# Supplementary material for: iMSC-mediated delivery of ACVR2B-Fc fusion protein reduces heterotopic ossification in a mouse model of fibrodysplasia ossificans progressiva
Source: Stem Cell Res Ther. 2024 Mar 18;15:83. doi: 10.1186/s13287-024-03691-7 (PMC10949803; doi:10.1186/s13287-024-03691-7)
Supplement: Supplementary file 1 — Additional file 1: Supplementary table 1. Reagents used in cell culture. Supplementary table 2. Primer sequences of genes in RT-qPCR. Supplementary table 3. Antibodies used in western blotting, IHC, and FACS analyses. [file 13287_2024_3691_MOESM1_ESM.docx]

| **Supplementary table 1: Reagents used in cell culture.** | | | | | |
| --- | --- | --- | --- | --- | --- |
| **Name** | **Company** | **Cat. No** | **Solvent for stocking** | **Stocking concentration** | **Working Concentration** |
| StemFit AK03N | Ajinomoto | / | / | / | / |
| StemFit Basic03 | Ajinomoto | / | / | / | / |
| SB-431542 | Selleck Chemicals | 301836-41-9 | DMSO | 25mM | 10 µM |
| CHIR99021 | Axon Medchem | 1386 | DMSO | 10mM | 1 µM |
| CultureSure Y-27632 | WAKO | 034-24024 | DMSO | 10mM | 10 µM |
| iMatrix-511 | Nippi | 892012 | / | / | 4.8 µg/well (6-well plate) |
| rhFGF2 | Wako | 060-04543 | 10% HSA 2.5ml + MilliQ | 20µg/ml | 20 ng/ml |
| rhEGF | R&D | CF 236-EG-200 | 10% HSA 2.5ml + MilliQ | 20µg/ml | 20 ng/ml |
| Fibronectin | Merck | FC010-10MG | / | / | 0.01 mg/ml |
| Recombinant Human TGF-β1 | R&D | 240-B-010 | 0.1% BSA/4mM HCL | 10μg/ml | 10 ng/ml |
| Recombinant Human TGF-β3 | R&D | 243-B3-200 | 0.1% BSA/4mM HCL | 10μg/ml | 10 ng/ml |
| Recombinant Human BMP-7 | R&D | 354-BP-010 | 0.1% BSA/4mM HCL | 100μg/ml | 100 ng/ml |
| Recombinant Human BMP-9 | R&D | 3209-BP-010 | 0.1% BSA/4mM HCL | 10μg/ml | 10 ng/ml |
| Recombinant Human Activin A | R&D | 338-AC-010 | 0.1% BSA/4mM HCL | 100μg/ml | 100 ng/ml |
| Cardiotoxin | latoxan | L8102 | PBS | 133.3uM | 13.33 uM |
| FK506 | medchemexpress | 104987-11-3 | DMSO | 1mM | 1 µM |

| **Supplementary table 2: Primer sequences of genes in RT-qPCR.** | | |
| --- | --- | --- |
| **Gene** | **Forward** | **Reverse** |
| β-Actin | CACCATTGGCAATGAGCGGTTC | AGGTCTTTGCGGATGTCCACGT |
| ACVR2B | CGATAGGCAGGAGTGTGTGG | GCAGAAGTTGCCTTCACAGC |
| ID1 | CCAACGCGCCTCGCCGGATC | CTCCTCGCCAGTGCCTCAG |
| CTGF | ACCCAACTATGATTAGAGCC | TTGCCCTTCTTAATGTTCTC |
| Luciferase | TACGTTAACAACCCCGAGGC | TCCACGATCTCCTTCTCGGT |

| **Supplementary table 3: Antibodies used in western blotting, IHC, and FACS analyses.** | | | | | | |
| --- | --- | --- | --- | --- | --- | --- |
| **Name** | **Company** | **Cat. No** | **Concentration** | **M.W ( kDa)** |  |  |
| SMAD2/3 (D7G7) XP® Rabbit mAb | Cell Signaling | #8685 | 1:1000 | 52, 60 |  |  |
| Phospho-SMAD2 (Ser465/467)/Smad3 (Ser423/425) (D27F4) Rabbit mAb | Cell Signaling | #8828 | 1:1000 | 52, 60 |  |  |
| Phospho-SMAD1/5/9 (D5B10) Rabbit mAb | Cell Signaling | #13820S | 1:1000 | 60 |  |  |
| Rabbit polyclonal to SMAD 1/5/8/9 | Abcam | ab13723 | 1:1000 | 51 |  |  |
| Rabbit polyclonal to Activin Receptor Type IIB/ACVR2B (aa 22-264) | Abcam | ab128544 | 1:1000 | 58 |  |  |
| Goat Anti-rabbit IgG, HRP-linked Antibody | Cell Signaling | 7074S | 1:2500 | / |  |  |
| Anti-β-Actin−Peroxidase antibody, Mouse monoclonal | Sigma-Aldrich | A3854 | 1:100000 | 42 |  |  |
| Collagen I alpha 1 Antibody (COL-1) | Novus Biologicals | NB600-450 | 1:100 | / |  |  |
| Anti-human Vimentin antibody | Abcam | ab16700 | 1:100 | / |  |  |
| Donkey anti-Rabbit IgG (H+L), Alexa Fluor® 488 | Invitrogen | A21206 | 1:100 | / |  |  |
| Goat anti-Mouse IgG (H+L), Alexa Fluor® 555 | Invitrogen | A21422 | 1:100 | / |  |  |
| APC anti-human CD44 | BioLegend | 338805 | 1:200 | / |  |  |
| APC anti-human CD73 | BioLegend | 344005 | 1:200 | / |  |  |
| APC Mouse Anti-Human CD90 | B&D | 559869 | 1:200 | / |  |  |
| APC CD105 (Endoglin) | Invitrogen | 17-1057-42 | 1:200 | / |  |  |
