# Supplementary material for: iMSC-mediated delivery of ACVR2B-Fc fusion protein reduces heterotopic ossification in a mouse model of fibrodysplasia ossificans progressiva
Source: Stem Cell Res Ther. 2024 Mar 18;15:83. doi: 10.1186/s13287-024-03691-7 (PMC10949803; doi:10.1186/s13287-024-03691-7)

Raw data of Western blot: Full, uncropped blot images

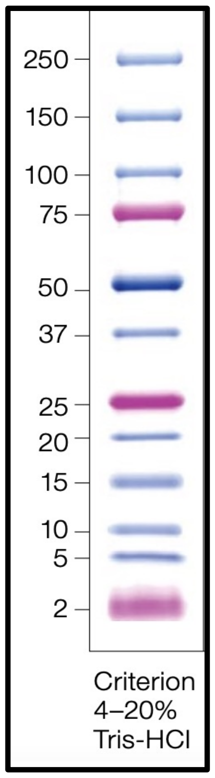

BIO-RAD #1610377

Protein Standards used in the study.

Figure 1D. Original western blots of ACVR2B in the iMSCs. β-actin was included as a loading control.

ACVR2B antibody: aa 22-264;
Constructed ACVR2B in plasmid: aa 19-134

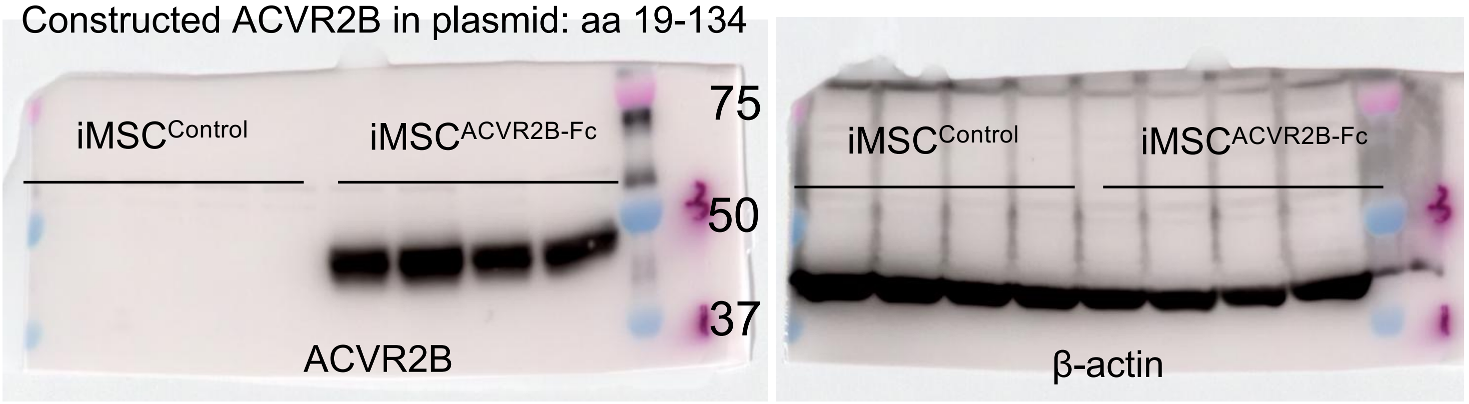

Figure 1G: Original western blots of p-SMAD1/5/8, SMAD1/5/8 and β-actin in the iMSCs treated with ligands.

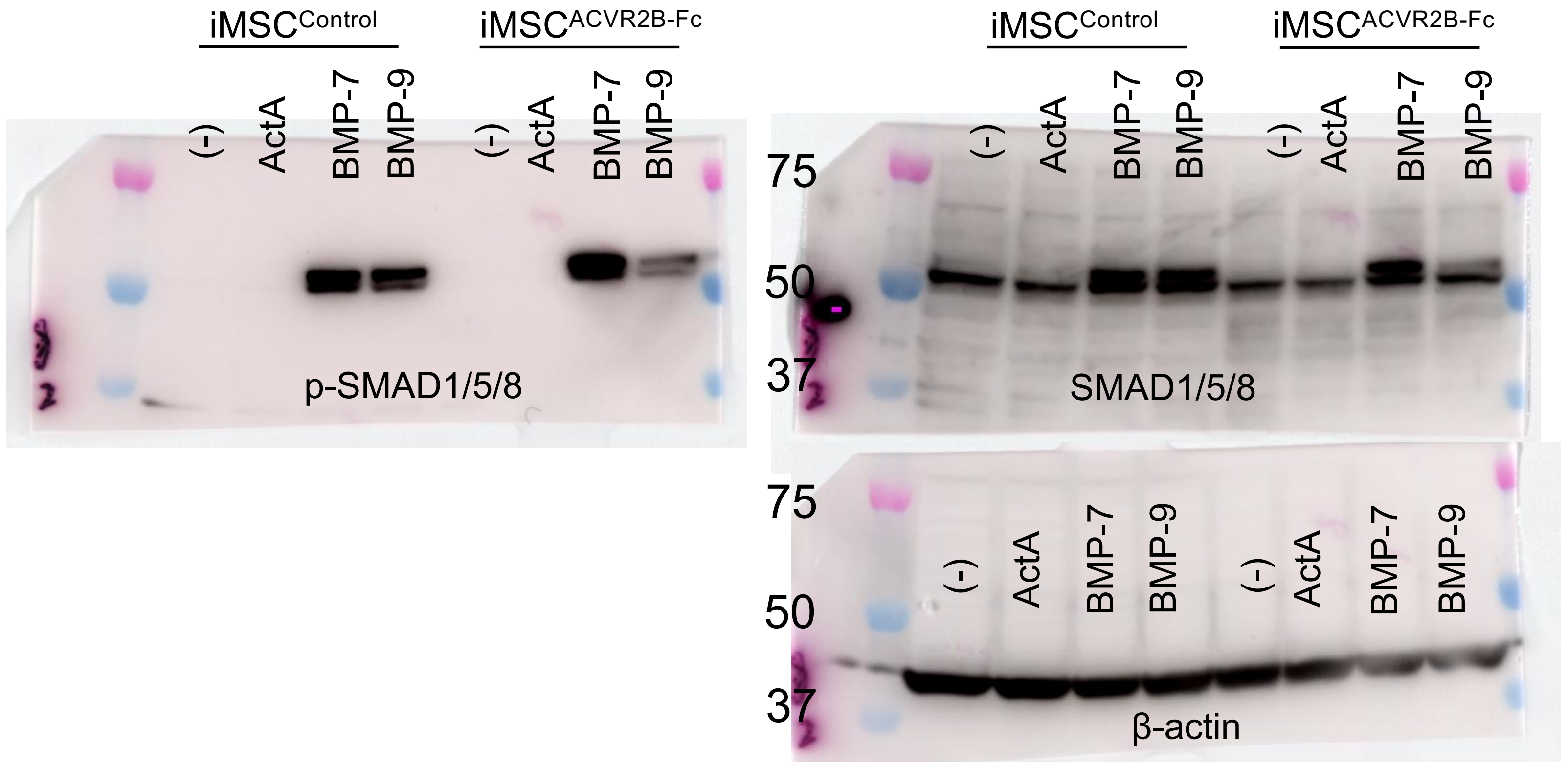

Figure 1J: Original western blots of p-SMAD2/3, SMAD2/3 and β-actin in the iMSCs treated with ligands.

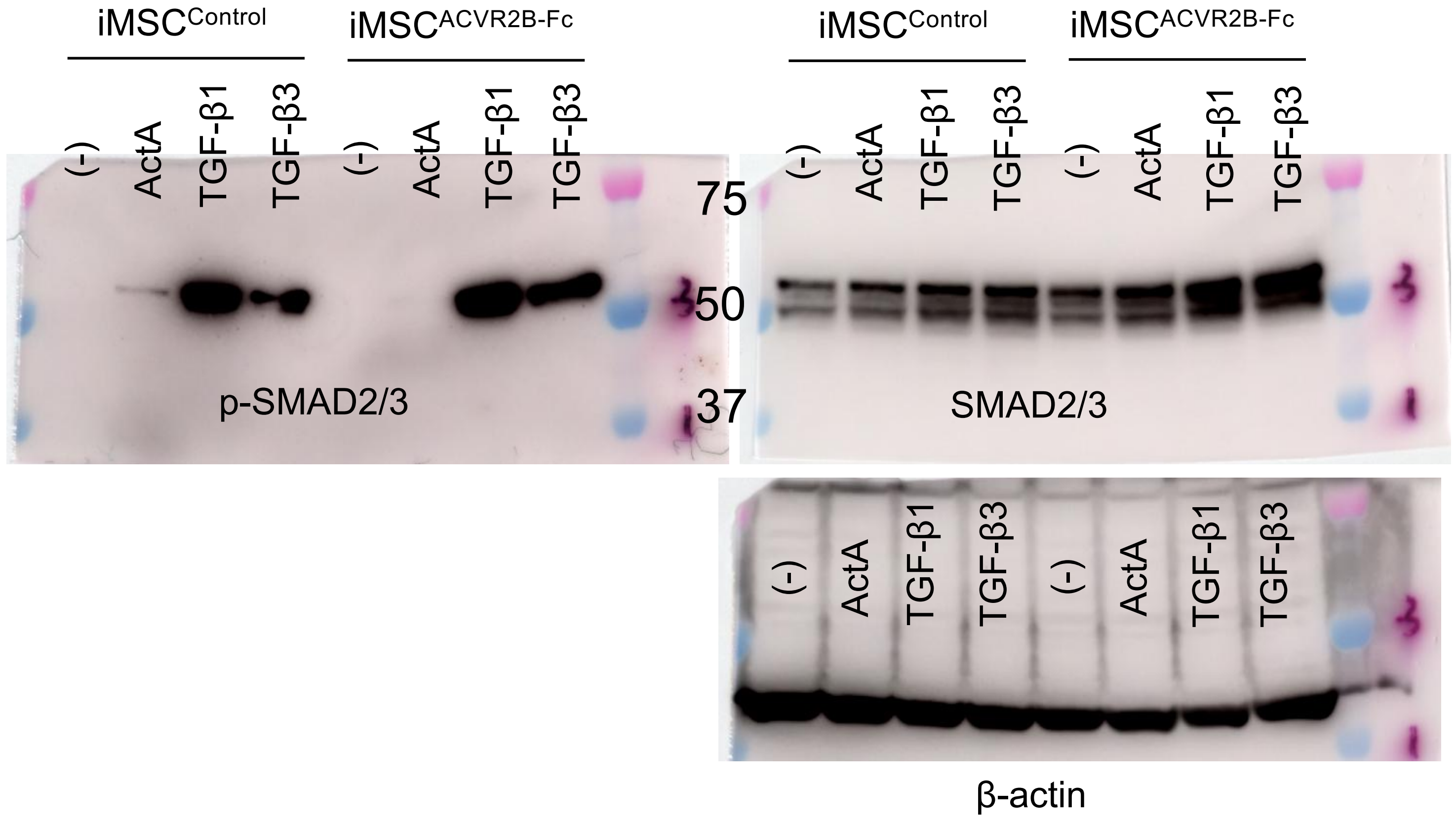

Figure 1L: Original western blots of p-SMAD1/5/8 and SMAD1/5/8 in the iMSCs treated with Activin-A or/and FK506.

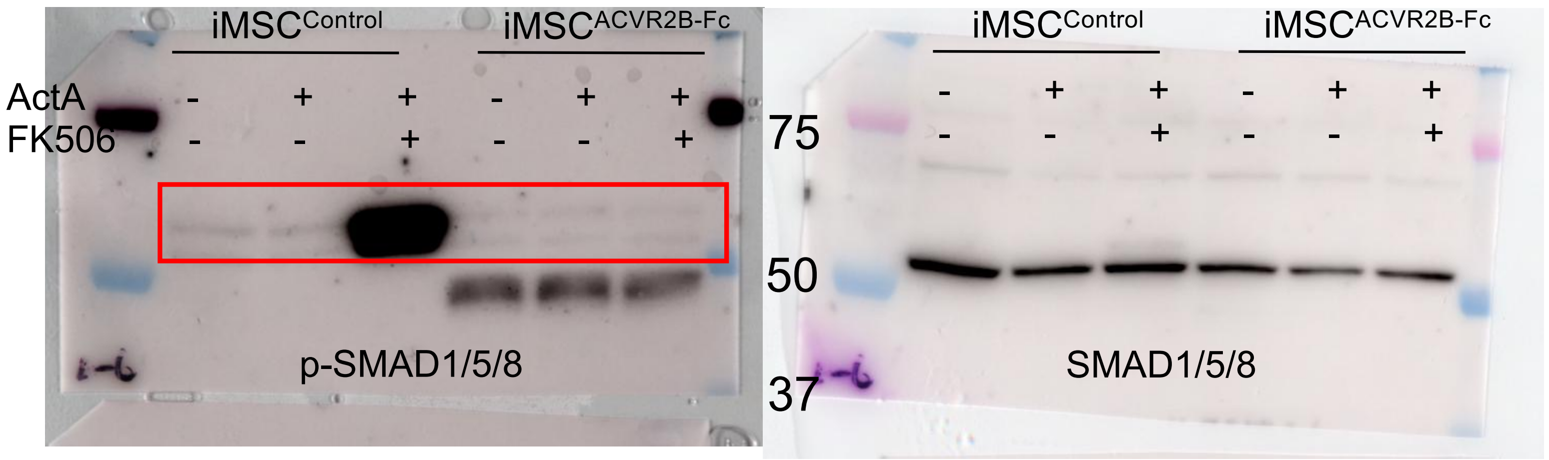

Figure1M: Original western blots of p-SMAD2/3 and SMAD2/3 in the iMSCs treated with Activin-A and FK506.

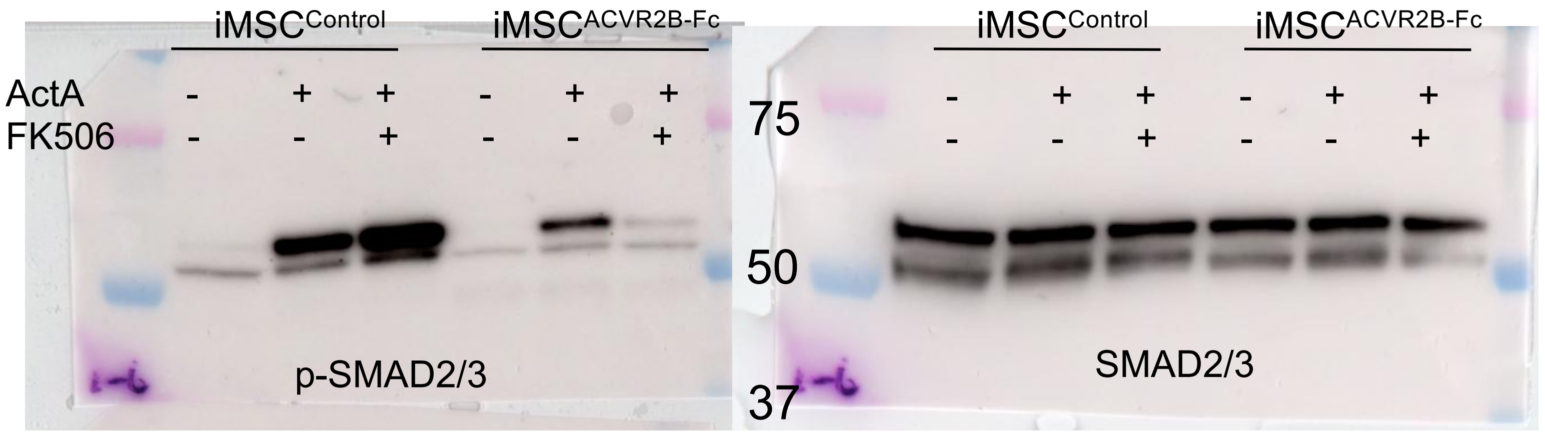

Figure 2E: Original western blots of p-SMAD1/5/8, SMAD1/5/8 and β-actin in the (res)FOP-iMSCs treated with ligands and ACVR2B-Fc conditioned medium.

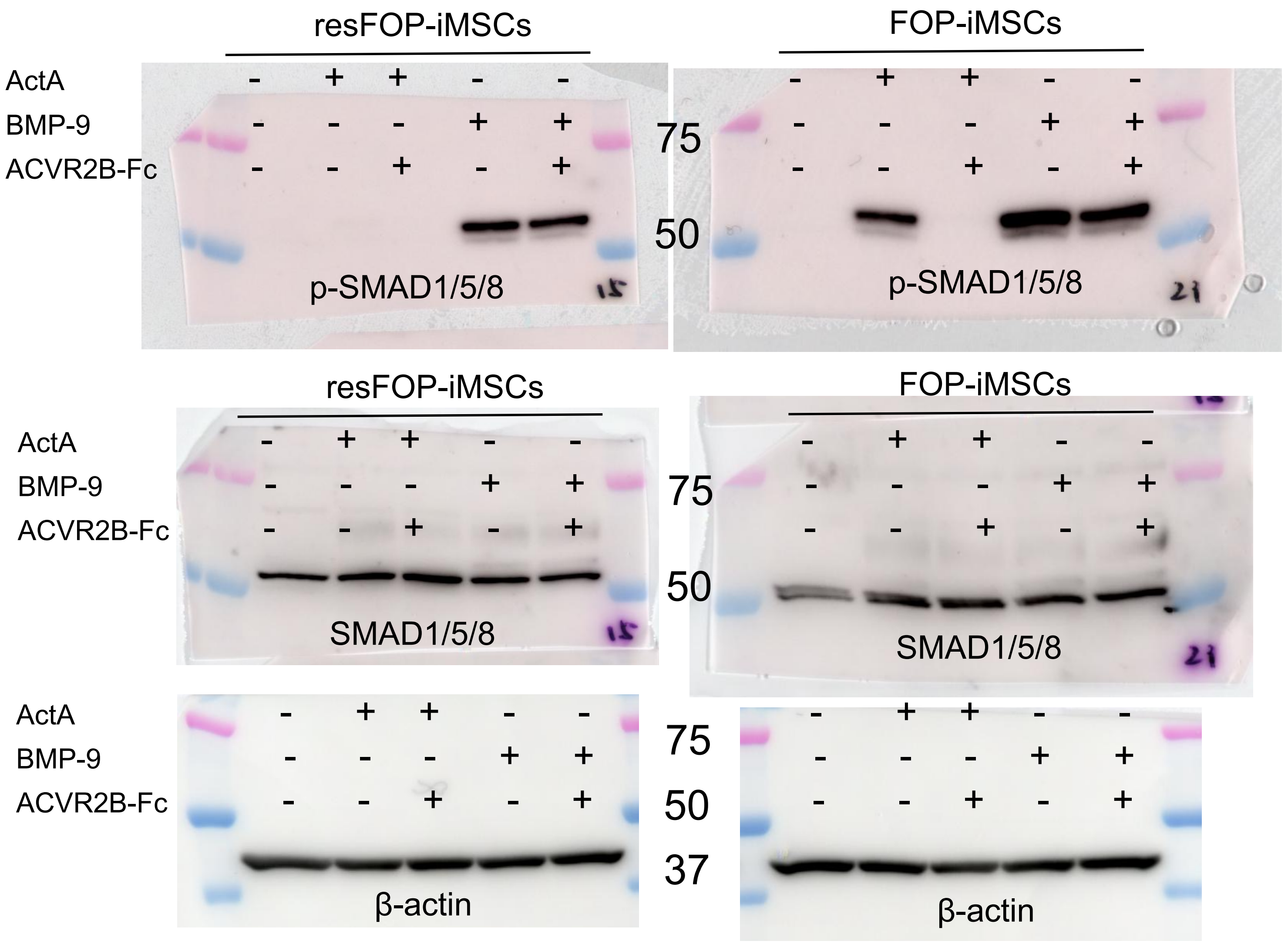

Figure 2I: Original western blots of p-SMAD2/3, SMAD2/3 and β-actin in the (res)FOP-iMSCs treated with Activin-A and ACVR2B-Fc conditioned medium.

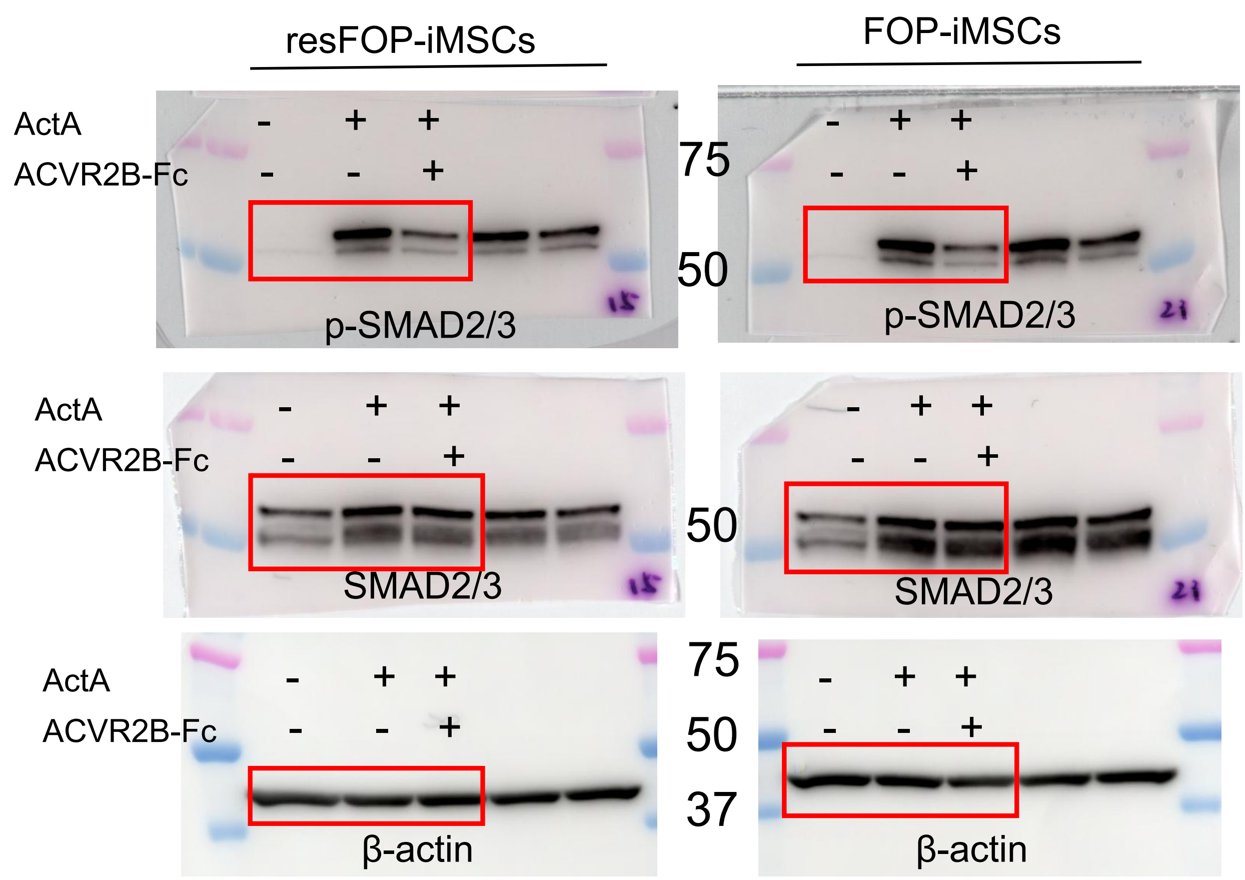

Figure S1E: Original western blots of ACVR2B in the iMSCs. β-actin was included as a loading control.

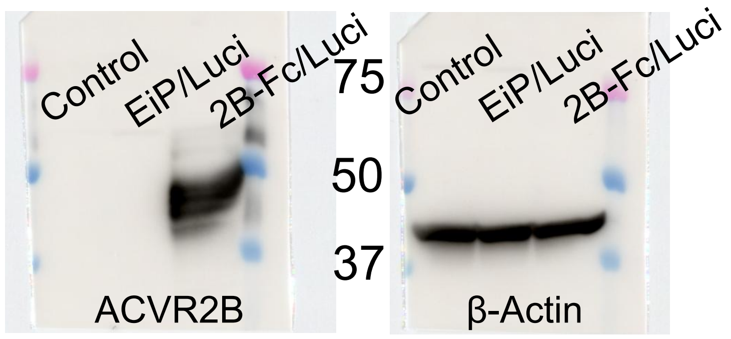

Supplement: Supplementary file 2 — Additional file 2: Raw data of Western blotting. [file 13287_2024_3691_MOESM2_ESM.pdf]
