# Supplementary figures and images for: iMSC-mediated delivery of ACVR2B-Fc fusion protein reduces heterotopic ossification in a mouse model of fibrodysplasia ossificans progressiva
Source: Stem Cell Res Ther. 2024 Mar 18;15:83. doi: 10.1186/s13287-024-03691-7 (PMC10949803; doi:10.1186/s13287-024-03691-7)

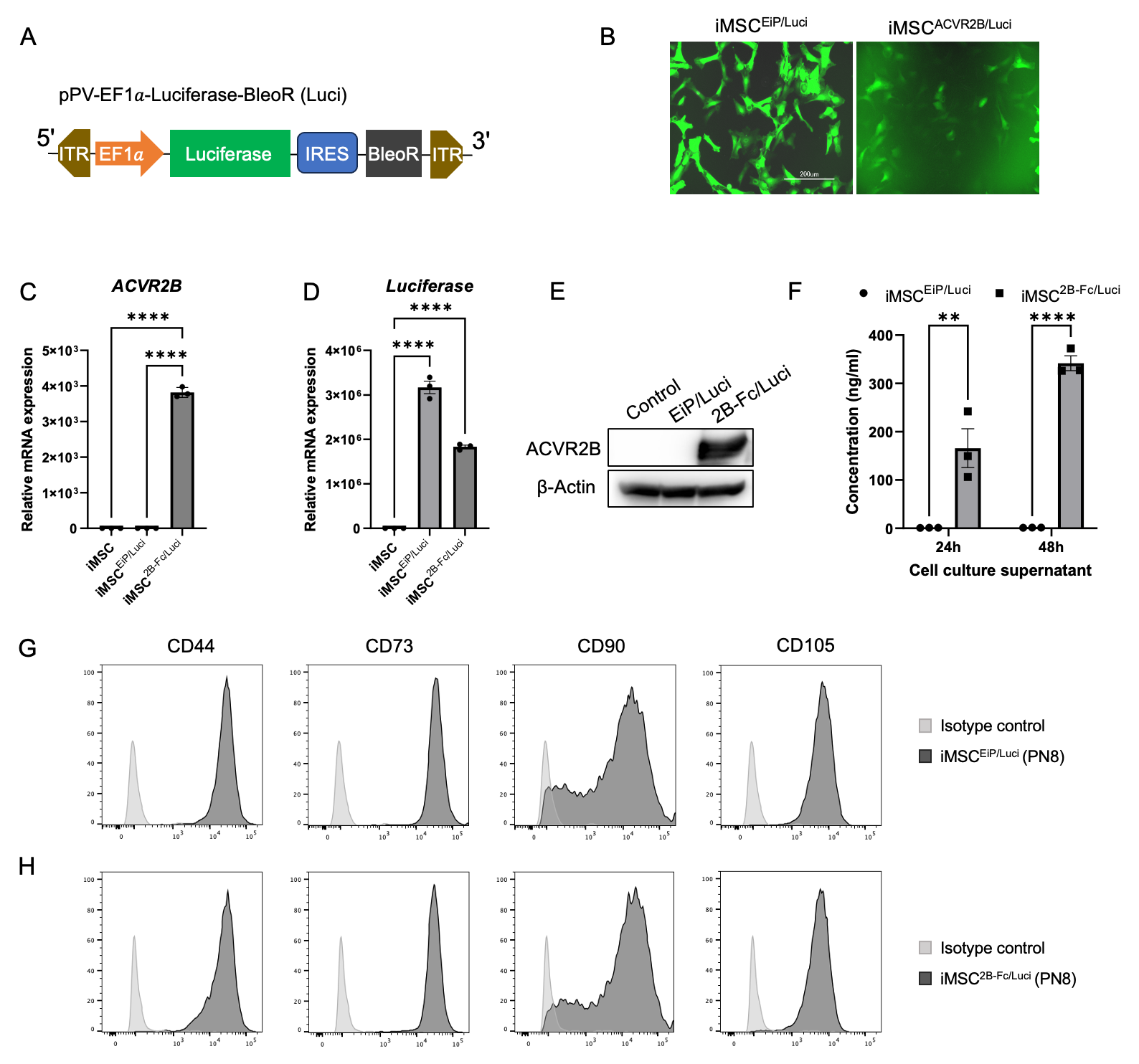

Supplement: Supplementary file 3 — Additional file 3: Fig. S1. Luciferase-expressing iMSCs. A Schematic linear maps of the luciferase plasmid used in the study. B Representative images of stably expressing cells after puromycin and zeocin selection for 7 d. iMSCEiP/Luci was used as a control. Scale bar, 200 µm. C, D The relative mRNA levels of ACVR2B and Luciferase in the iMSC2B-Fc/Luci and iMSCEiP/Luci cells based on qPCR analysis. E Protein levels of ACVR2B in the iMSC2B-Fc/Luci and control cells based on western blotting analysis. β- was used as an internal reference. All full-length blots are presented in Additional file 1: Fig. S1E. F The concentration of His-tag proteins in the cell supernatants at two-time points based on ELISA. G, H The expression of MSC-related markers in iMSCEiP/Luci and iMSC2B-Fc/Luci (dark gray) and isotype control (gray) cells. Results represent the mean ± SEM. **, P < 0.01; ****, P < 0.0001 by one-way ANOVA with Turkey’s multiple comparison test for qPCR and two-way ANOVA with Šídák's multiple comparisons test for ELISA. [file 13287_2024_3691_MOESM3_ESM.tiff]

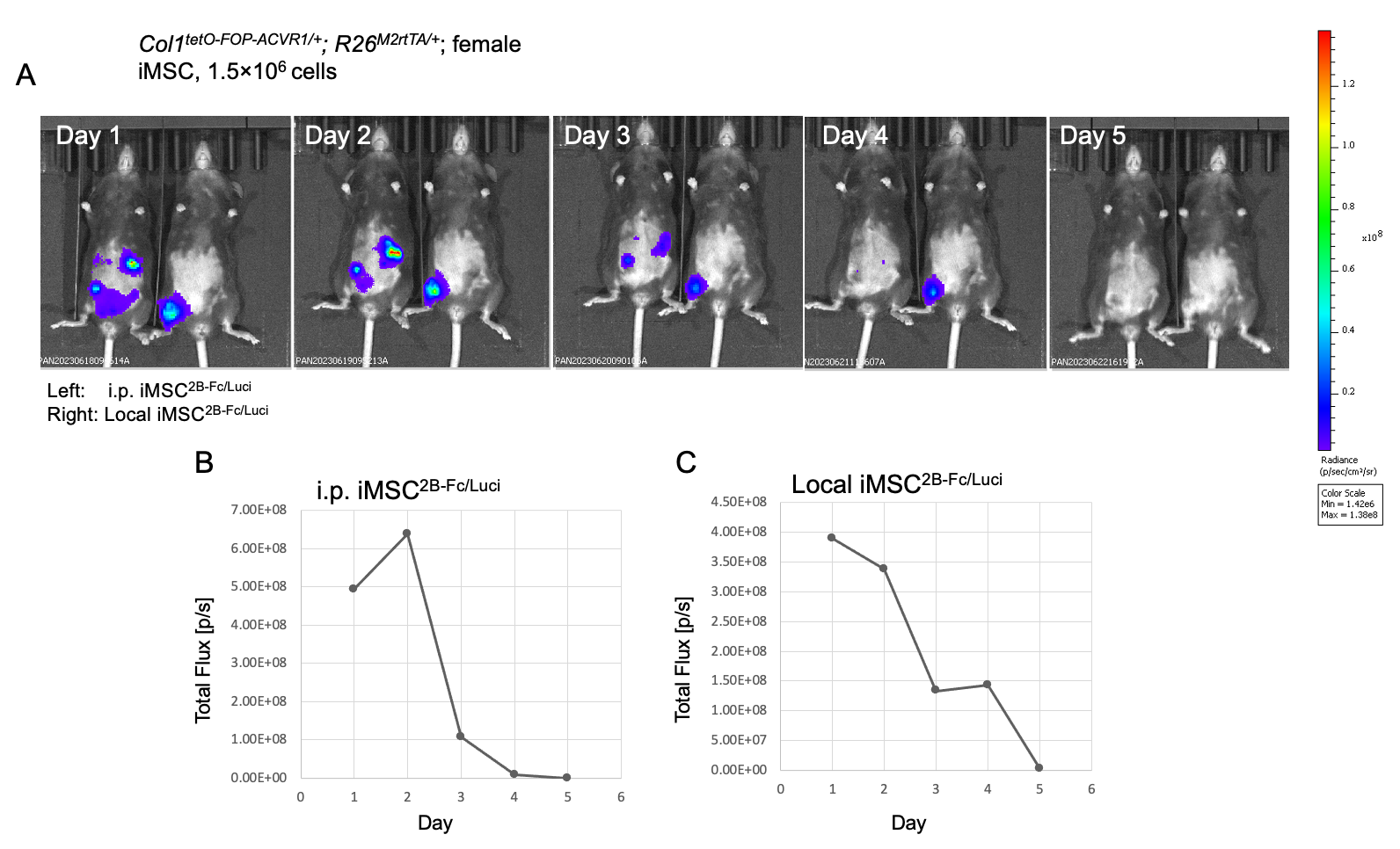

Supplement: Supplementary file 4 — Additional file 4: Fig. S2. Tracking of donor cells after intraperitoneal and local transplantation. A In vivo imaging of donor cells within 5 d. CTX was injected into the right gastrocnemius muscle to initiate muscle injury. For the left mouse, cells were transplanted intraperitoneally. For the right mouse, cells were transplanted locally on the CTX-injected site. B, C The time course of the luciferase signal intensity of two administrations. The signal disappeared on day 5. [file 13287_2024_3691_MOESM4_ESM.tiff]

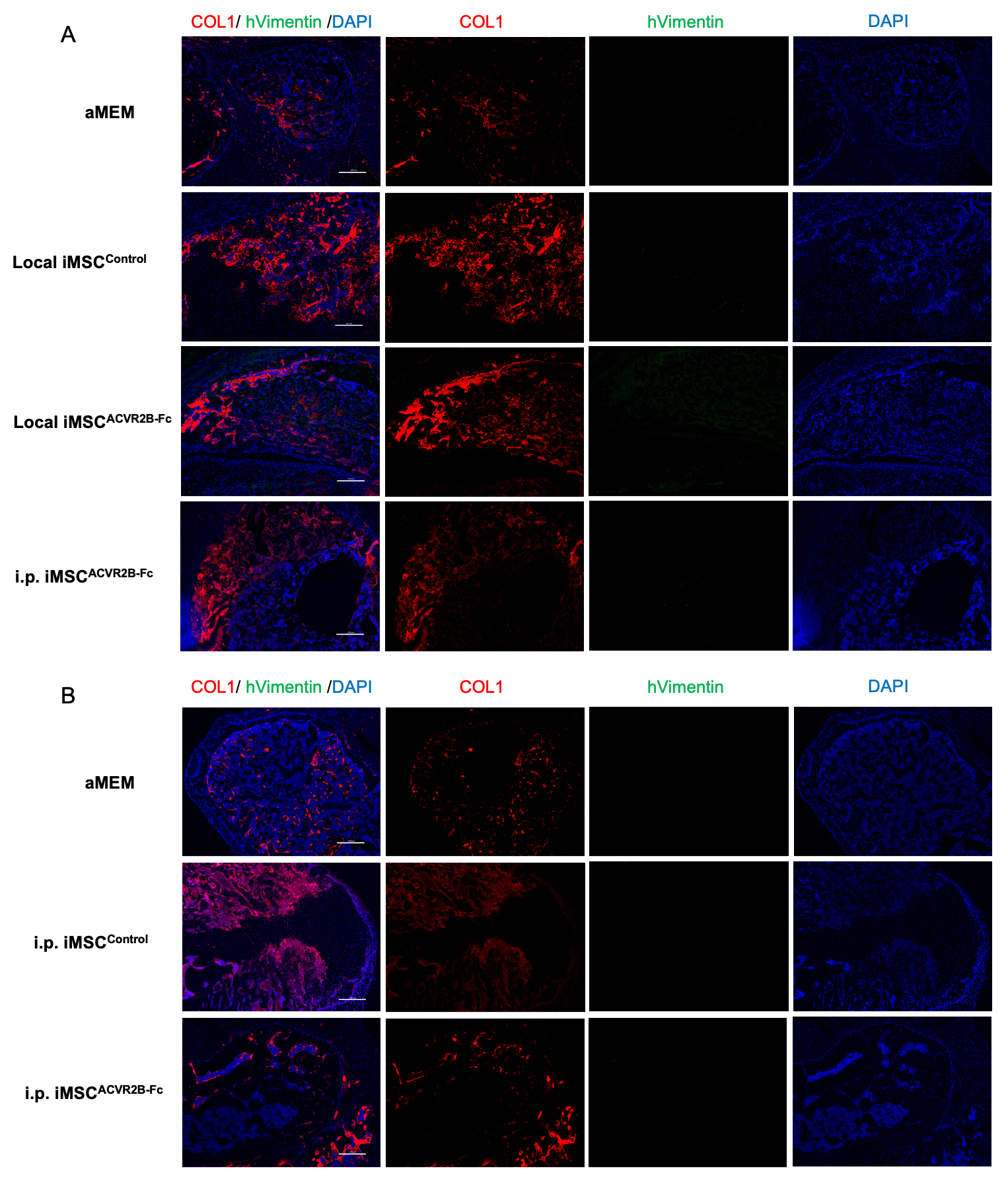

Supplement: Supplementary file 5 — Additional file 5: Fig. S3. Overlay of the immunofluorescence staining. A Co-staining of COL1, hVimentin, and DAPI in Figure 4, lane 4. B Co-staining of COL1, hVimentin, and DAPI in Figure 6, lane 4. Scale bar, 200 µm. [file 13287_2024_3691_MOESM5_ESM.tiff]
